# Selective electrochemical generation of benzylic radicals enabled by ferrocene-based electron-transfer mediators†
†Electronic supplementary information (ESI) available: Experimental details, procedures and spectroscopic characterisations. See DOI: 10.1039/c7sc04032f


**DOI:** 10.1039/c7sc04032f

**Published:** 2017-11-06

**Authors:** Alastair J. J. Lennox, Jordan E. Nutting, Shannon S. Stahl

**Affiliations:** a Department of Chemistry , University of Wisconsin–Madison , 1101 University Avenue , Madison , Wisconsin 53706 , USA . Email: stahl@chem.wisc.edu

## Abstract

The generation and intermolecular functionalisation of carbon-centred radicals has broad potential synthetic utility. Herein, we show that benzylic radicals may be generated electrochemically from benzylboronate derivatives at low electrode potentials (*ca.* –0.3 V *vs.* Cp_2_Fe^0/+^) *via* single electron oxidation. Use of a catalytic quantity of a ferrocene-based electron-transfer mediator is crucial to achieve successful radical functionalisation and avoid undesirable side reactions arising from direct electrochemical oxidation or from the use of stoichiometric ferrocenium-based oxidants.

## Abstract 2

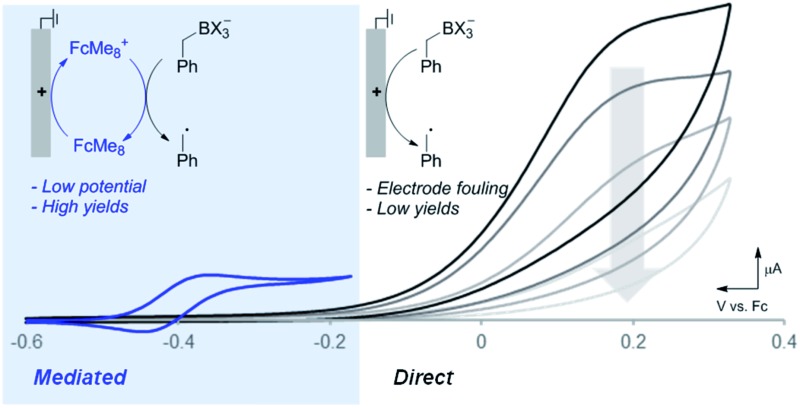
The use of ferrocene mediators offers significant advantages over direct electrolysis in the generation and functionalization of radicals from benzylboronates.

## 

Carbon-centred radicals are versatile reaction intermediates,^1^,^2^ and recent studies have led to numerous methods to exploit these species in unique synthetic transformations.^3^ Radical pathways can be lower in energy and provide different selectivity relative to those based on other reactive carbonaceous species, such as carbanions or carbocations. The growing interest in accessing radical-based pathways for organic synthesis motivates efforts toward the development of new methods to generate these species.

Electrochemistry provides a unique opportunity to generate and manipulate radicals due to its reagent-free and tunable control over redox processes, and it continues to expand as a powerful technology for organic synthesis.^4^ The oxidative generation and functionalisation of radicals is often intimately linked to the nature of the chemical oxidant employed.^5^ Electrochemical oxidation of radical precursors, however, is not linked to the subsequent radical functionalisation step, thus potentially providing the basis for a wider variety of intermolecular functionalisation strategies. There are myriad examples of electrochemical oxidation to access net two-electron reactivity,^6^ but far fewer electrochemical methods exist that rely on single-electron pathways to selectively generate and functionalise neutral radicals (Fig. 1A). Most precedents feature trapping of an electrochemically generated radical by O_2_,^7^ while those undergoing anaerobic functionalisation are scarce.^8^ The limited number of precedents may be attributed, in part, to the proclivity of carbon-centred radicals to undergo side reactions when generated in close proximity to an electrode surface. Common side reactions include direct reaction with the electrode, further oxidation of the radical to afford carbocation species, and homocoupling of the radicals to afford dimeric (Kolbe-type) products (Fig. 1B). Intramolecular functionalisation of radicals can circumvent some of these problems, and a number of demonstrations of such reactivity have been recently described.^9^ Ultimately, however, it would be desirable to control the intermolecular reactivity of electrochemically generated radicals. Herein, we show that significantly improved control of radical reactivity is possible through the use of ferrocene-based electron-transfer mediators. The mediator shuttles redox equivalents into the bulk solution, away from the electrode surface where the radical is susceptible to degradation pathways, and it enables productive intermolecular reactivity through an electrochemical-chemical (EC′) mechanism (Fig. 1C). The results herein illustrate the utility of a mediated electrolysis strategy for radical generation,^10^,^11^ with potentially broader implications for the growing field of electro-organic chemistry.

**Fig. 1:**
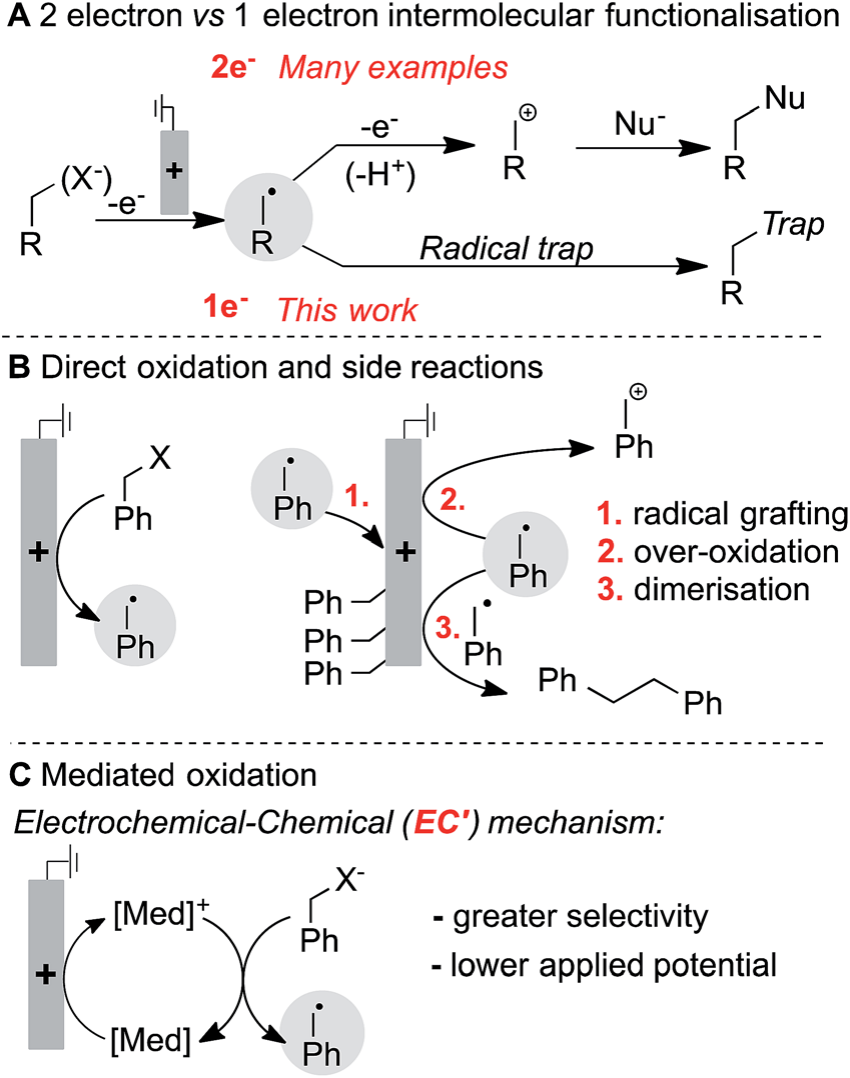
Aspects of electrochemical radical generation.

Benzylboronic esters were selected as appealing entry-points for electrochemical generation of carbon-centred radicals. The oxidative liberation of radicals from organoboron compounds has been demonstrated under photochemical^12^ conditions and with stoichiometric oxidants,^13^ but electrochemical oxidation of organoboron reagents to generate radical intermediates has yet to be fully explored.^14^

Benzylpinacol boronic ester (**1a**) was analysed by cyclic voltammetry, and was found not to undergo oxidation within the examined potential window (Fig. 2A). This observation is consistent with the need to use strong chemical oxidants (*ca.* 2 V *vs.* NHE)^15^ to oxidise neutral boronic acids.13a,b Addition of NaOH to **1a** generates the anionic boronate (11B NMR), which is readily oxidised at lower potentials. The cyclic voltammogram (CV) exhibits an irreversible redox wave,^16^ suggesting single electron-transfer (SET) at the anode forms an unstable neutral radical that rapidly homolyses *via* C–B bond cleavage to give the benzylic radical (Fig. 2B). Variation of the boron substituents led to significant changes in the boronate oxidation potential, with an observed potential range of nearly 1 V (Fig. 2C). The potential is affected by both the ancillary ligation (*e.g.*, diolate, diamide, trifluoro) and the identity of the anionic activator (X^–^), which appears to include both electronic (*cf.* TBAF *vs.* NaOH for **1a**) and steric effects (*cf.* KOt-Bu *vs.* KOMe). The resulting benzylboronate species exhibit redox potentials that are more than 0.5 V lower than many functional groups commonly assumed to be easily oxidised, (*e.g.*, enamines (0.0–0.2 V *vs.* Fc/Fc^+^), trialkylamines (0.45–0.55 V) or anilines (0.1–0.6 V)).^17^

**Fig. 2:**
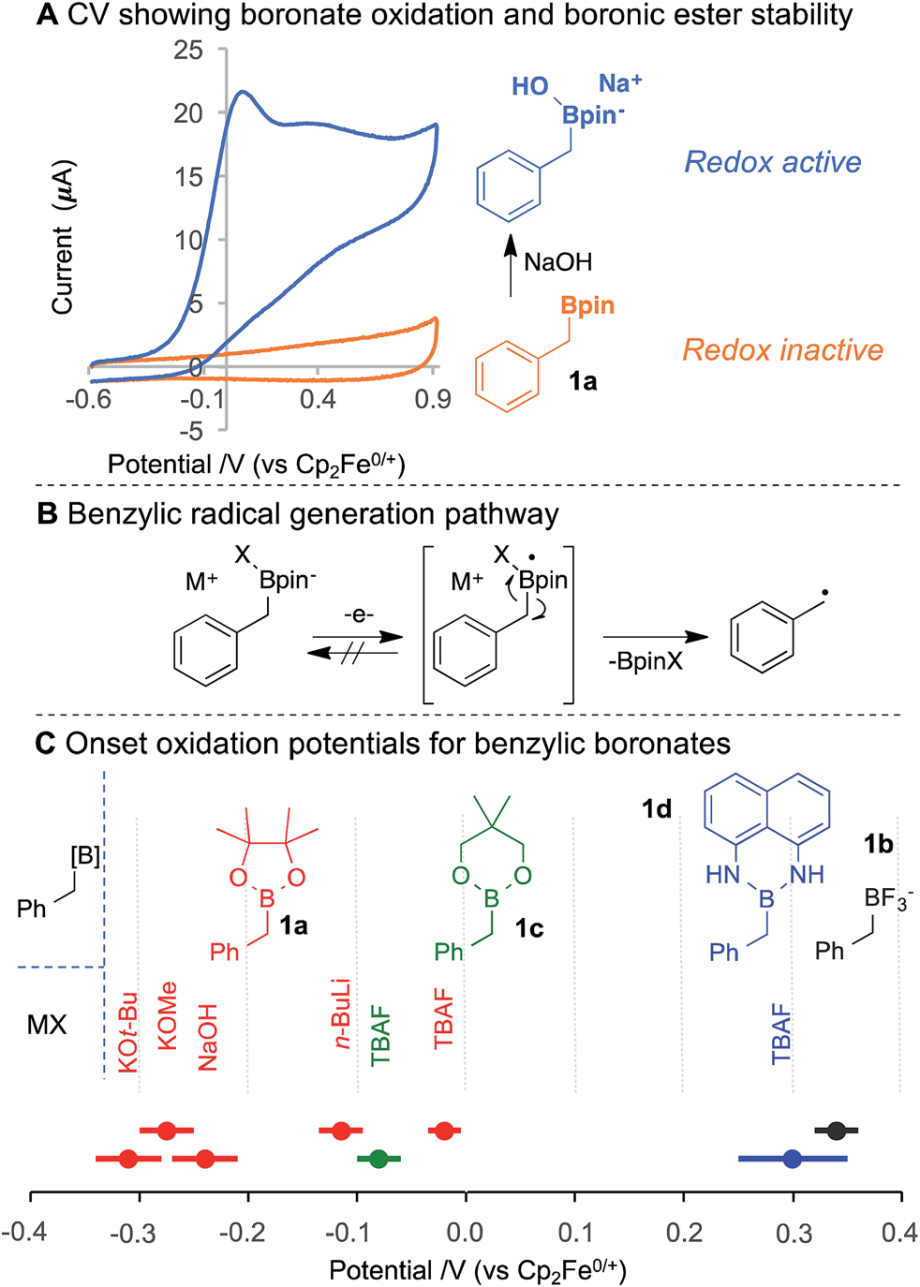
(A) CVs showing no oxidation of **1a** without added base. (B) Single electron oxidation of benzylboronate with homolytic C–B bond cleavage to reveal radical. (C) Onset oxidation potentials measured for a variety of boronic esters (**1a**, **1c**, **1d**) with bases (colour-coded for their use with each boronic ester) and boronate **1b**. Lines through points indicate a range of uncertainty.

Benzyltrifluoroborate **1b** oxidises at the highest potential, consistent with the anionic stabilisation from three electronegative fluorides. The fluoride adduct of boronic ester **1c** (derived from addition of TBAF) is oxidised more readily than the fluoride adduct of **1a**, suggesting the 6-membered ring of **1c** engenders a less stable tetrahedral boronate. In the absence of an anionic activator, aniline oxidation was observed in diaminoboron reagent **1d** at approximately 0.1 V *vs.* Fc/Fc^+^. Addition of TBAF, however, increases the oxidation potential and leads to an irreversible CV trace,^18^ which indicates boronate oxidation and C–B bond cleavage. No electrochemical activity was observed for benzyl MIDA boronates and ill-defined redox activity was observed for a benzyl cyclic triol boronate.^18^ The significant influence of anion ligation to boronic ester derivatives offers a flexible strategy to adjust the oxidation potential for radical generation, and may find useful synthetic and materials applications beyond those presented herein.

During the voltammetric studies, cycling the applied potential multiple times led to a decrease in the magnitude of the response current (Fig. 3). This effect was observed for all tested boronates, with both glassy-carbon (GC) and Pt disk electrodes,^18^ and it was not attenuated by the presence of an exogenous radical trap, which could plausibly compete for reaction with the benzylic radical. The activity could only be restored after polishing the electrodes. SEM analysis of the electrode surface before and after fouling did not reveal bulk changes,^18^ suggesting that electrical insulation arises from molecular scale modification of the electrode surface.^19^ This conclusion is consistent with precedents for intentional derivatisation of electrode surfaces *via* oxidation of benzylcarboxylates^19^ or reduction of diazonium reagents.^20^

**Fig. 3:**
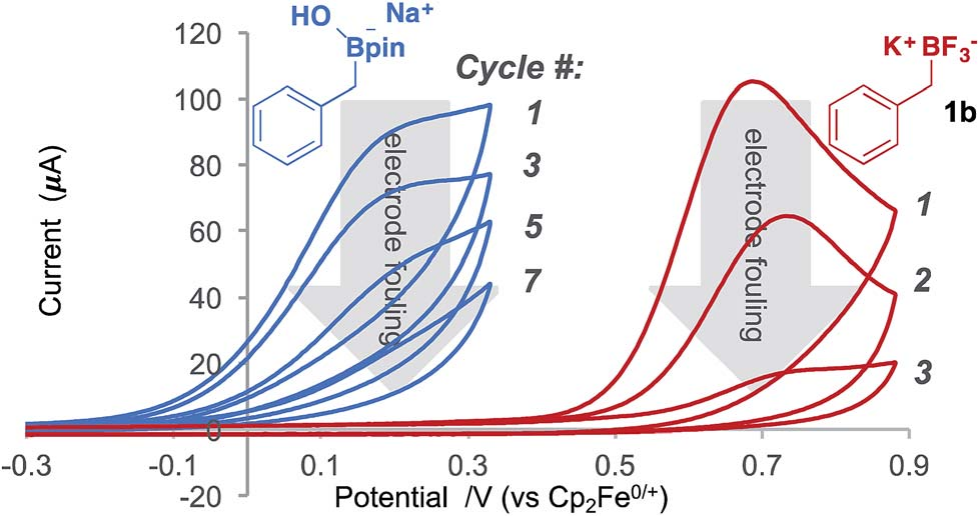
Cycled CVs reveal a current loss due to benzylic radical grafting and subsequent electrode fouling. CVs of **1a** + NaOH in MeCN and TBAP (0.1 M) and oxo-TEMPO (5 mM) (blue) and **1b** (5 mM) with dihydroanthracene (5 mM) in MeCN and TBAP (0.1 M) (red).

The electrode fouling observed by voltammetry was also manifested in the oxidation of boronates *via* bulk electrolysis (Scheme 1). The electrolysis was performed with RVC in the presence of 4 equivalents of TEMPO to trap the benzylic radical. The TEMPO-functionalised product was observed, but only in moderate yields and with a relatively poor mass balance (MB).^21^ This outcome, which could not be improved by altering the identity of the boronate, is attributed to non-productive substrate consumption and electrode fouling.

**Scheme 1:**
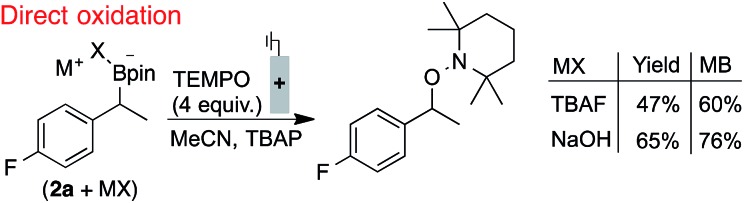
Inefficient radical generation and functionalisation with direct oxidation. Reactions performed in divided cells under N_2_ atmosphere with RVC:Pt electrodes (0.1 mmol scale), under constant potential (0.0 V, TBAF) or current (0.4 mA, NaOH), NMR yields shown. **2a** used in bulk electrolyses for ^19^F NMR probe. Secondary benzylboronate oxidation potentials are between 60–90 mV lower than the primary benzylboronates shown in Fig. 2.^18^

These observations prompted us to consider the use of an electrochemical mediator. Triarylamines^22^ and imidazoliums^23^ have been reported as electrochemical single-electron redox mediators, however, they operate at much higher oxidation potentials (*ca.* 0.5–1.5 V) that are poorly matched to the low potential benzylboronates (Fig. 2). On the other hand, ferrocene (Fc) derivatives display redox potentials in the appropriate range. Ferrocene itself was recently demonstrated by Xu and co-workers as an electrochemical mediator in radical generation for intramolecular functionalisation,^24^ but other ferrocene derivatives have yet to be explored in this role. The redox states of all tested ferrocene derivatives show stable and reversible activity (CV), and thus we decided to investigate their use as catalytic mediators for boronate oxidation.

Voltammetric analysis of two ferrocene derivatives, octamethyl–ferrocene (FcMe_8_) and dibromo-ferrocene (FcBr_2_), displayed an increased oxidation current in the presence of a boronate substrate (Fig. 4). This current increase is typical of an electrochemical-chemical (EC′) mechanism (Fig. 1C), in which the mediator is regenerated on the timescale of the CV scan, and is proportional to catalyst activity.^25^

**Fig. 4:**
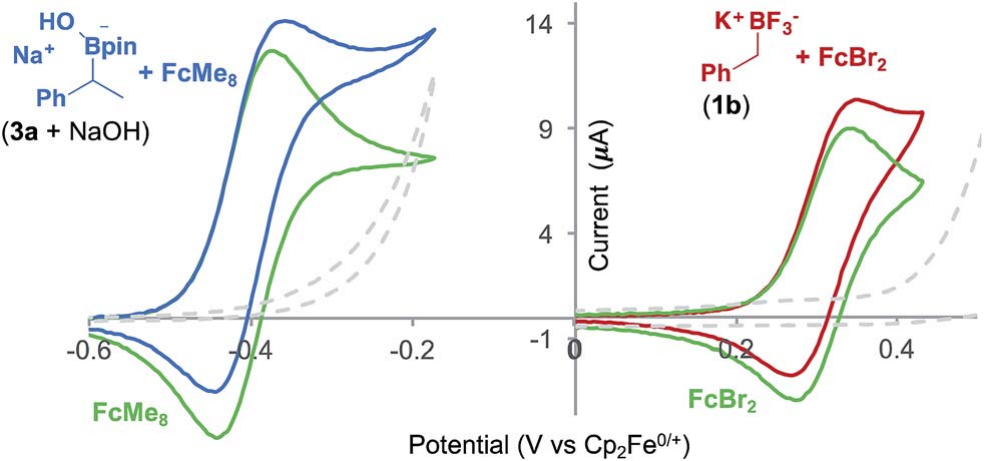
CVs showing ferrocene derivative mediated boronate oxidation. The increase in the current of the ferrocene derivative oxidation is due to catalytic boronate oxidation. Left: CVs (average of 3 runs) of FcMe_8_ (1.5 mM) in MeCN and TBAP (0.1 M), 10 mV s^–1^ (green) and, added to that, (1-phenethyl)pinacol boronic ester (50 mM) and NaOH (50 mM) (blue). Any current due to background substrate (3 + NaOH (50 mM)) oxidation (grey dashed) has been removed from the blue catalysis trace. Right: CVs (average of 3 runs) of FcBr_2_ (1 mM) in MeCN:THF (1 : 1) and TBAP (0.1 M) 10 mV s^–1^ (green) and, added to that, **1b** (5 mM) (red). Current due to background substrate (**1b** (5 mM)) oxidation (grey dashed) has been removed from the red catalysis trace.

The onset redox potentials of these two ferrocene derivatives are approximately 200 mV lower than the onset potential of the respective boronates (3a + NaOH and **1b**, respectively). This feature is designed to attenuate direct substrate oxidation at the electrode and ensure that the majority of the substrate is oxidised in the bulk solution by the mediator. The thermodynamically uphill electron transfer (200 mV = 4.6 kcal mol^–1^) is driven by rapid and irreversible C–B bond homolysis from the oxidised boronate derivative. Employing less oxidising ferrocene derivatives, in which the energy difference is larger, led to a decrease in the magnitude of the catalytic current, as evident by CV.^18^

The utility of ferrocenium mediators for boronate oxidation was then probed under bulk electrolysis conditions (Scheme 2).^26^ Use of a catalytic quantity of FcMe_8_ (10 mol%) led to a significantly improved yield of the benzylic TEMPO adduct. With constant current electrolysis, the oxidation proceeds at a lower potential (*ca.* 200 mV) than in the absence of the mediator, which attenuates electrode fouling processes that otherwise consume substrate (Fig. 1B). A lower concentration of TEMPO could also be tolerated under these conditions.

**Scheme 2:**
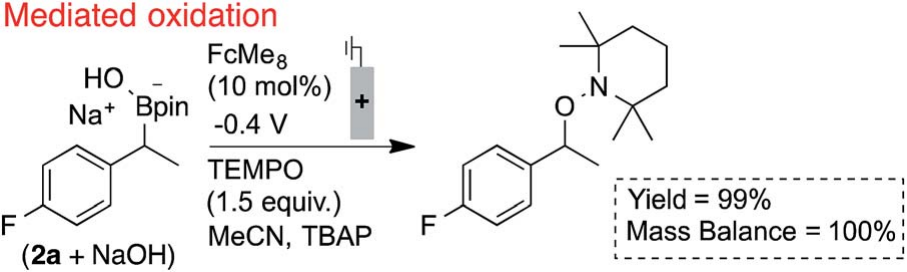
Efficient radical generation and productive functionalisation facilitated by the inclusion of a catalytic electron mediator. Reactions performed in divided cells under N_2_ atmosphere with RVC:Pt electrodes (0.1 mmol scale), NMR yields shown.

Electrochemically regenerating a catalytic ferrocenium derivative proved to be more effective than employing a stoichiometric quantity of the oxidant. The pairing of FcMe_8_^+^ or FcBr_2_^+^ with low and high potential boronates (**2a** + NaOH and **2b**), respectively, only afforded low yields of the desired coupled products (Scheme 3). The increased concentration of the Fc^+^-based oxidants led to over-oxidation byproducts and boronate decomposition.^18^,^27^,^28^ These observations show that controlled electrochemical regeneration of a catalytic mediator can have advantages over the use of a stoichiometric chemical oxidant. The effectiveness of the mediated electrochemical oxidation strategy proved successful with other low-potential benzylboronates, exhibiting high yields and mass balances in each case (Scheme 4). This product class is useful^29^ as, for example, cation precursors^30^ or as initiators for controlled nitroxide-mediated polymerisation reactions.^31^

**Scheme 3:**
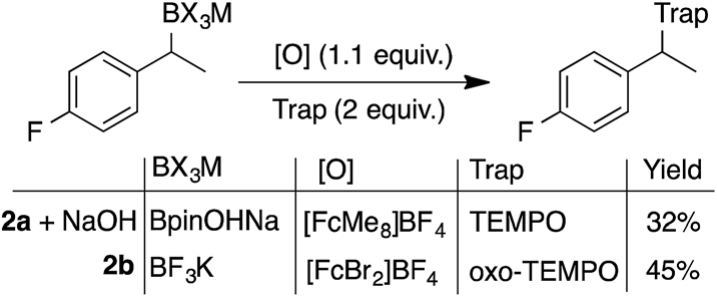
Inefficient radical generation and functionalisation with the use of stoichiometric quantities of oxidant. The more oxidatively resilient oxo-TEMPO was required when used in combination with the more oxidising FcBr_2_^+^.

**Scheme 4:**
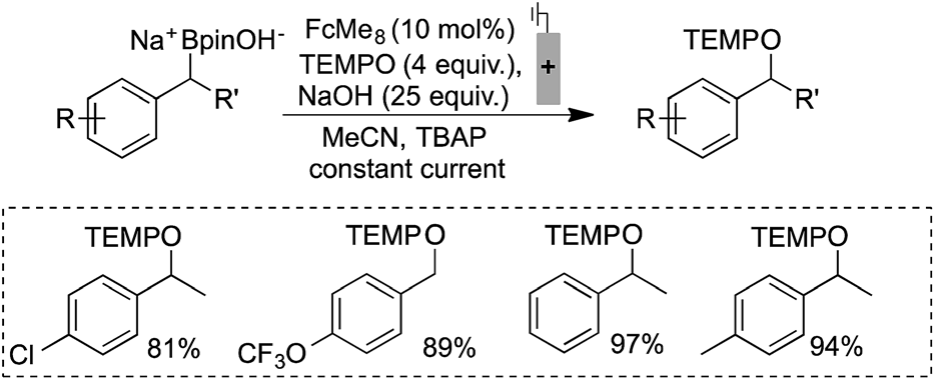
Efficient electrochemical mediated single electron oxidation and trapping of benzylic radical demonstrated. Reactions performed in divided cells under N_2_ atmosphere with RVC:Pt electrodes (0.1 mmol scale, 0.4 mA), NMR yields shown.

In summary, this study demonstrates the benefits of catalytic redox mediators in the electrochemical oxidative conversion of benzyl boronates to benzylic radicals. Mediated electrolysis avoids electrode fouling and side-product formation, which occur during direct electrochemical oxidation. Mediated electrolysis also offers several advantages over the use of stoichiometric ferrocenium-based oxidants, which lead to over-oxidation and substrate decomposition. These insights should aid the development of electrochemical methods for the generation and intermolecular functionalisation of carbon-centred radicals, a potentially transformative strategy in synthetic chemistry.

## Conflicts of interest

There are no conflicts to declare.

## Supplementary Material

Supplementary information
