# Supplementary material for: Selective electrochemical generation of benzylic radicals enabled by ferrocene-based electron-transfer mediators
Source: Chem Sci. 2017 Nov 6;9(2):356–61. doi: 10.1039/c7sc04032f (PMC5909123; doi:10.1039/c7sc04032f)
Supplement: Supplementary file 1 [file SC-009-C7SC04032F-s001.pdf]

Electronic Supplementary Material (ESI) for Chemical Science.
This journal is © The Royal Society of Chemistry 2017

Selective electrochemical generation of benzylic radicals enabled by ferrocene-based electron-transfer mediators

Alastair J. J. Lennox, Jordan E. Nutting, and Shannon S. Stahl

Department of Chemistry, University of Wisconsin-Madison, 1101 University Avenue, Madison, Wisconsin 53706, United States

Table of contents:

1. General experimental details S2
2. Oxidation potential study of boronates S4
3. CV studies of electrode fouling S8
4. SEM analysis of electrode fouling S10
5. CV studies of mediated boronate oxidation S14
6. Bulk electrolyses S17
7. Reactions with stoichiometric oxidant S19
8. Synthesis & characterization of TEMPO-trapped products S20
9. Synthesis of benzylic boron compounds S22
10. Synthesis of benzylic pinacol boronic esters S23
11. Spectra S24
12. References S32

1. General experimental details

Reagents:

Organic solvents were obtained from an LC Technology Solutions Inc. solvent purification system and purged for 15-20 min with N_2 gas before use. All commercially available organic compounds were used as received unless otherwise specified. 1,1'-Ferrocene dibromide, ferrocene, ferrocene bromide, 1,1'-ferrocene diacetate, 1,1'-dimethylferrocene, octamethylferrocene, potassium benzyltrifluoroborate (**1**), 4-(Trifluoromethoxy)benzylboronic acid pinacol ester (**2g**), benzylboronic acid MIDA ester, TBAF, *n*-BuLi, KO*t*-Bu, KOMe, NaOH, benzyl pinacol boronic ester (**1b**), and tetrabutylammonium perchloride were purchased from Sigma Aldrich. 4,4,5,5-Tetramethyl-2-[(4-trifluoromethoxy)benzyl]-1,3,2-dioxaborolane was purchased from Combi-Blocks. Benzylic pinacol boronic esters (**2b**-**2f**),[1] benzyl neopentylglycol boronic ester (**1c**),[2] benzyl diaminoboronamide (**1e**)[2] and dibromoferrocenium tetrafluoroborate[3] were prepared according to literature procedures.

Instruments and techniques:

^1H, ^{13}C, and ^{19}F NMR spectra were recorded on Bruker 400 and 500 MHz spectrometers. Chemical shifts are given in parts per million (ppm) relative to residual solvent peaks in the ^1H and ^{13}C NMRs and relative to $CFCl_3$ in the ^{19}F NMR. High-resolution mass spectra were obtained using a Thermo Q ExactiveTM Plus by the mass spectrometry facility at the University of Wisconsin. Chromatographic purification of products was accomplished by chromatography on Silicycle P60 silica gel (particle size 40-63 μm, 230-400 mesh) using Teledyne Isco Combiflash R_f or Biotage Isolera One flash chromatography systems. Thin-layer chromatography (TLC) was performed on Silicycle silica gel UV254 pre-coated plates (0.25 mm). Visualization of the developed chromatogram was performed by using UV lamps and $KMnO_4$ stain.

All cyclic voltammetric, chronoamperometric and chronopotentiometric measurements were performed at room temperature using a Pine WaveNow PGstat. The CV experiments were carried out in a three-electrode cell configuration with a glassy carbon (GC) working electrode (3 mm diameter, unless otherwise stated) and a platinum wire counter electrode. The potentials were measured versus an $Ag/AgNO_3$ (0.01 M) reference electrode (all electrodes from BASi) and recorded against a ferrocene/ferrocenium reference. The GC working electrode was polished with alumina before each experiment. Bulk electrolysis experiments were performed in a custom-built inert atmosphere divided cell with a Schlenk tap attached. Reticulated vitreous carbon (RVC) was used for the working electrode material, a platinum wire for the counter electrode, and $Ag/AgNO_3$ (0.01 M) for the reference electrode.

Electrochemical cells:

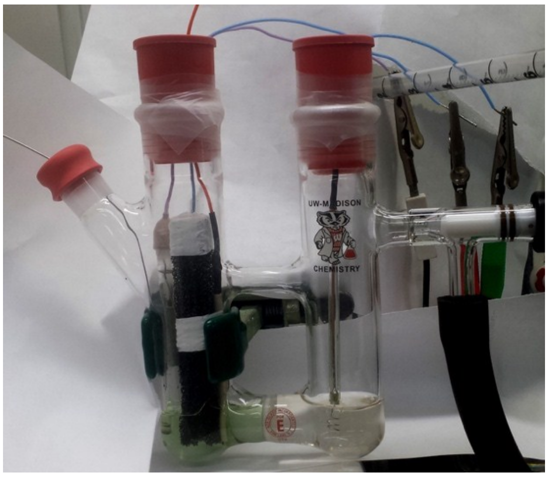

Figure S1*:* Electrolysis cell used for anaerobic electrolyses. Cell is equipped with a gas/vacuum line inlet, cathodic cell separated from the anodic solution by a porous glass frit.

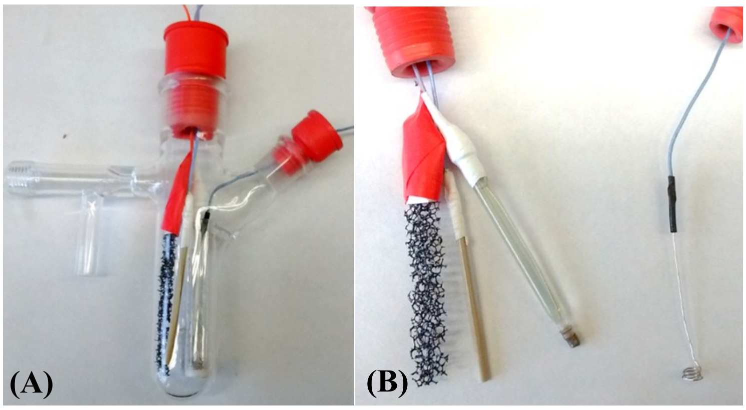

Figure S2: A: Electrolysis cell used for anaerobic electrolyses and **B**: electrodes for the electromediated oxidation of benzylic boronates. Cell is equipped with a gas/vacuum line inlet, and low volume cathodic cell separated from the anodic solution by a porous glass frit.

Abbreviations:

GC = glassy carbon
TBAP = tetrabutylammonium perchlorate
TBAF = tetrabutylammonium fluoride
CV = cyclic voltammetry
CA = chronoamperometry
WE = working electrode
Fc = Ferrocene

2. Oxidation potential study of boronates

Titration studies

A variety of bases were added in half equivalent amounts to boronic esters in MeCN and TBAP. After each addition, a sample (0.5 mL) was removed and analysed by ^{11}B NMR, while the remaining solution was analysed by cyclic voltammetry.

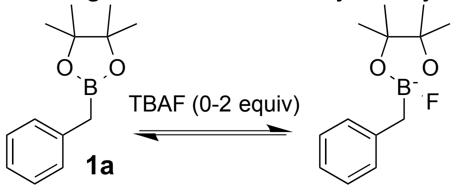

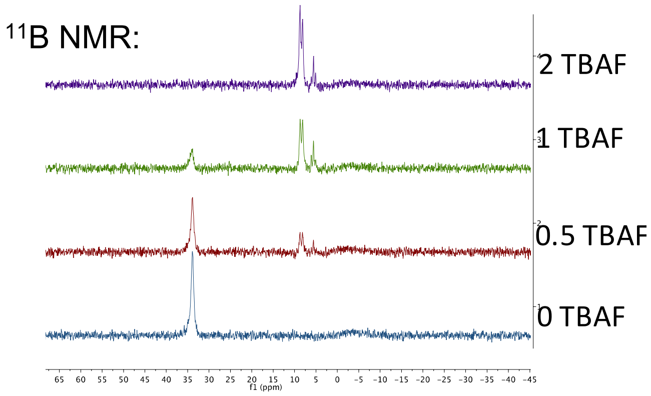

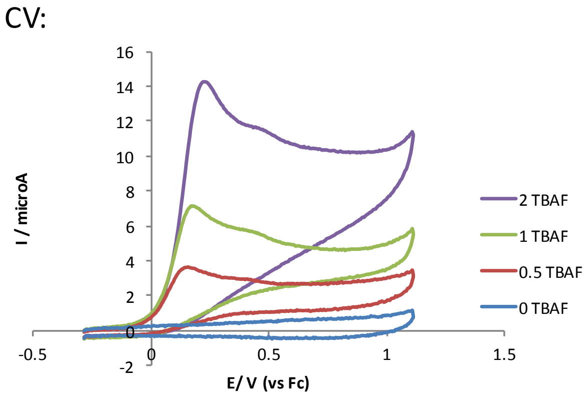

Figure S3. Left: ^{11}B NMR of titration of TBAF into **1a**; Right: CV trace after each addition of TBAF. **1a** (1 mM), TBAF (1 M in THF), 50 mV/s, WE = GC, Ref = Ag/AgNO$_3$, MeCN, TBAP (100 mM).

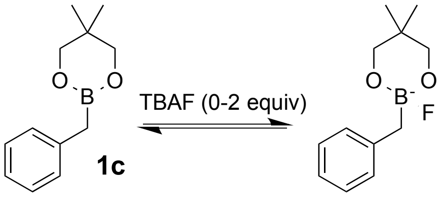

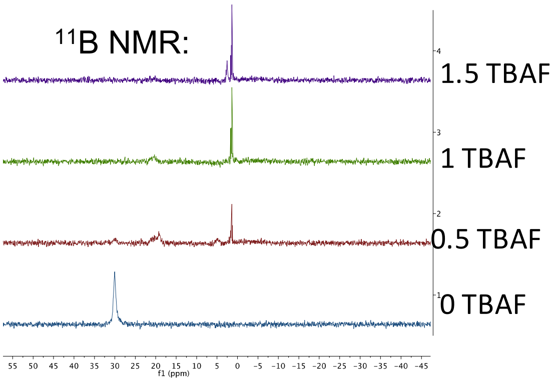

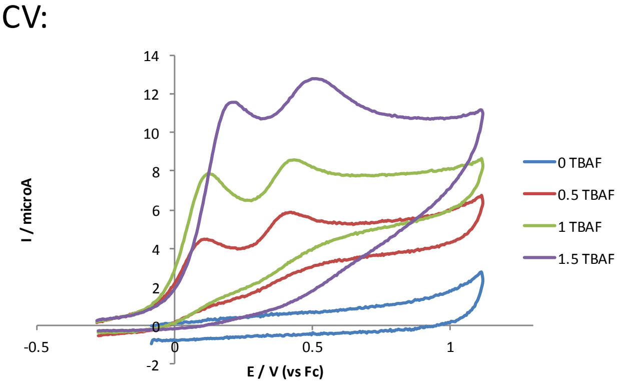

Figure S4. Left: ^{11}B NMR of titration of TBAF into **1c**; Right: CV trace after each addition of TBAF. **1c** (1 mM), TBAF (1 M in THF), 50 mV/s, WE = GC, Ref = Ag/AgNO$_3$, MeCN, TBAP (100 mM).

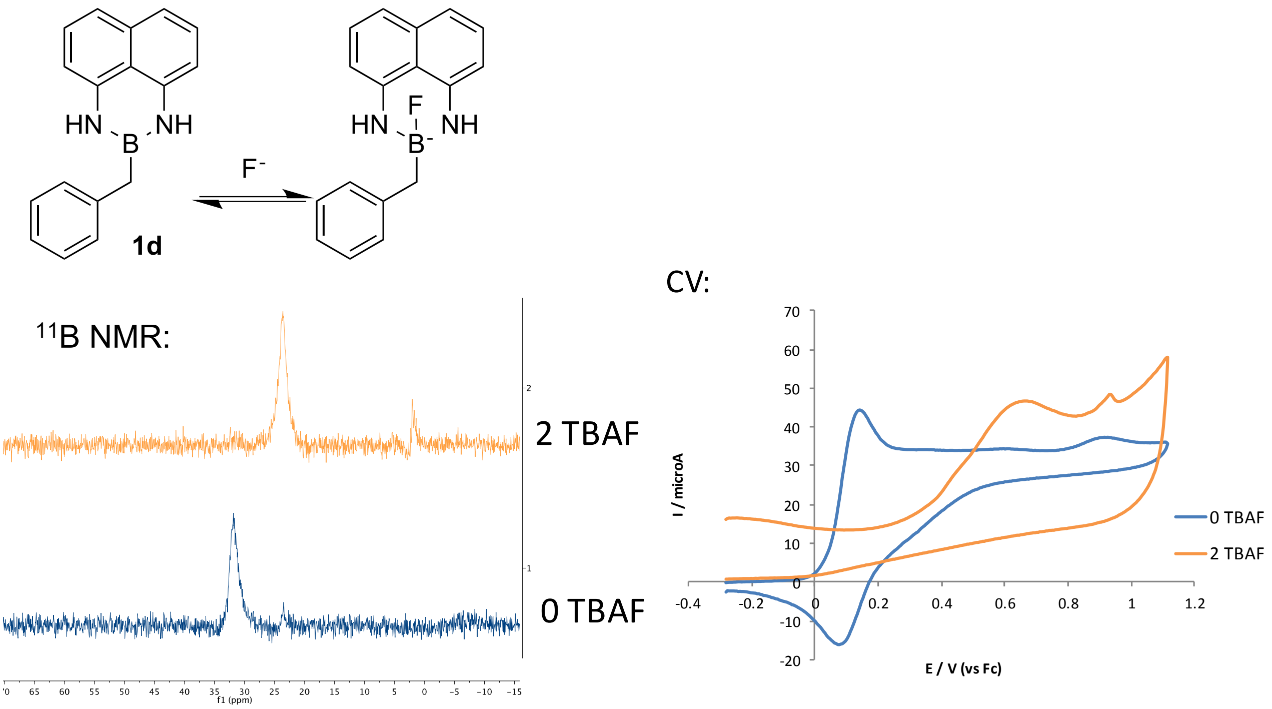

Figure S5: Left: ^{11}B NMR of titration of TBAF into **1d**; Right: CV trace after each addition of TBAF. **1d** (1 mM), TBAF (1 M in THF), 50 mV/s, WE = GC, Ref = Ag/AgNO$_3$, MeCN, TBAP (100 mM).

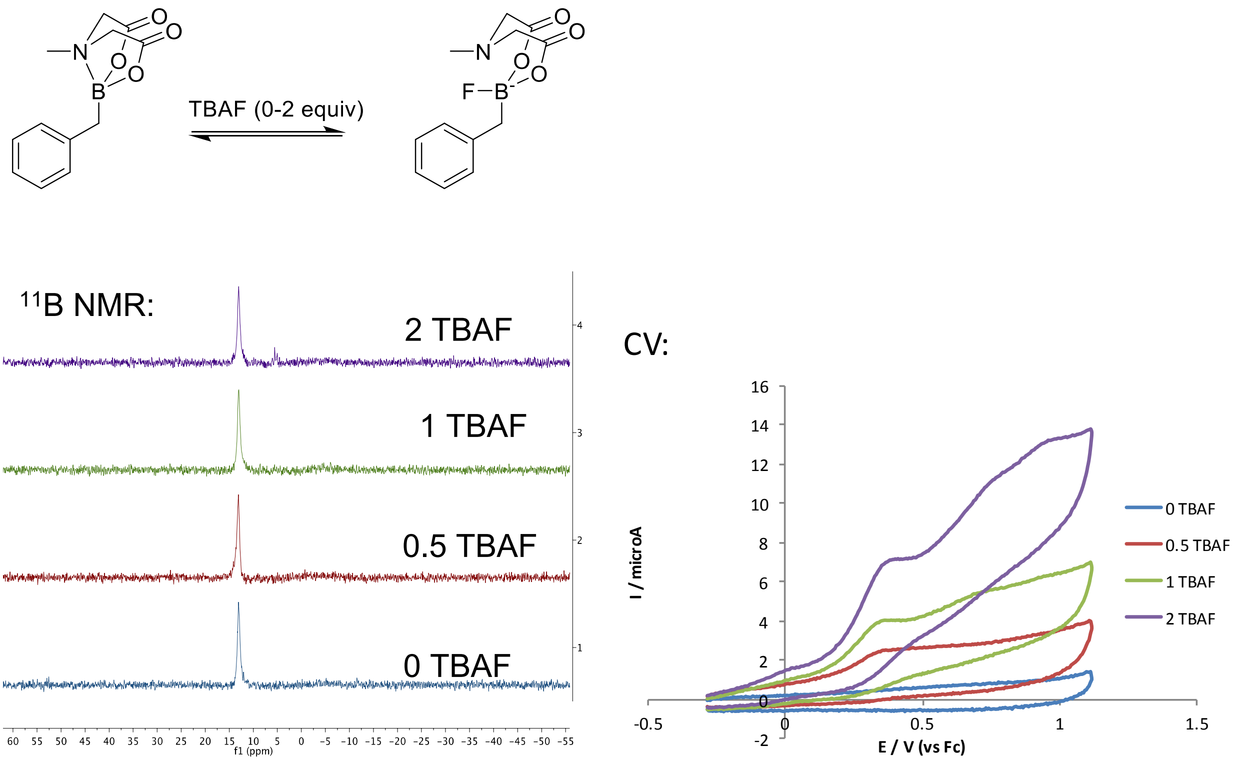

Figure S6: Left: ^{11}B NMR of titration of TBAF into benzyl MIDA boronate; Right: CV trace after each addition of TBAF. Benzyl MIDA boronate (1 mM), TBAF (1 M in THF), 50 mV/s, WE = GC, Ref = Ag/AgNO$_3$, MeCN, TBAP (100 mM). The current observed is the same as that observed from TBAF alone.

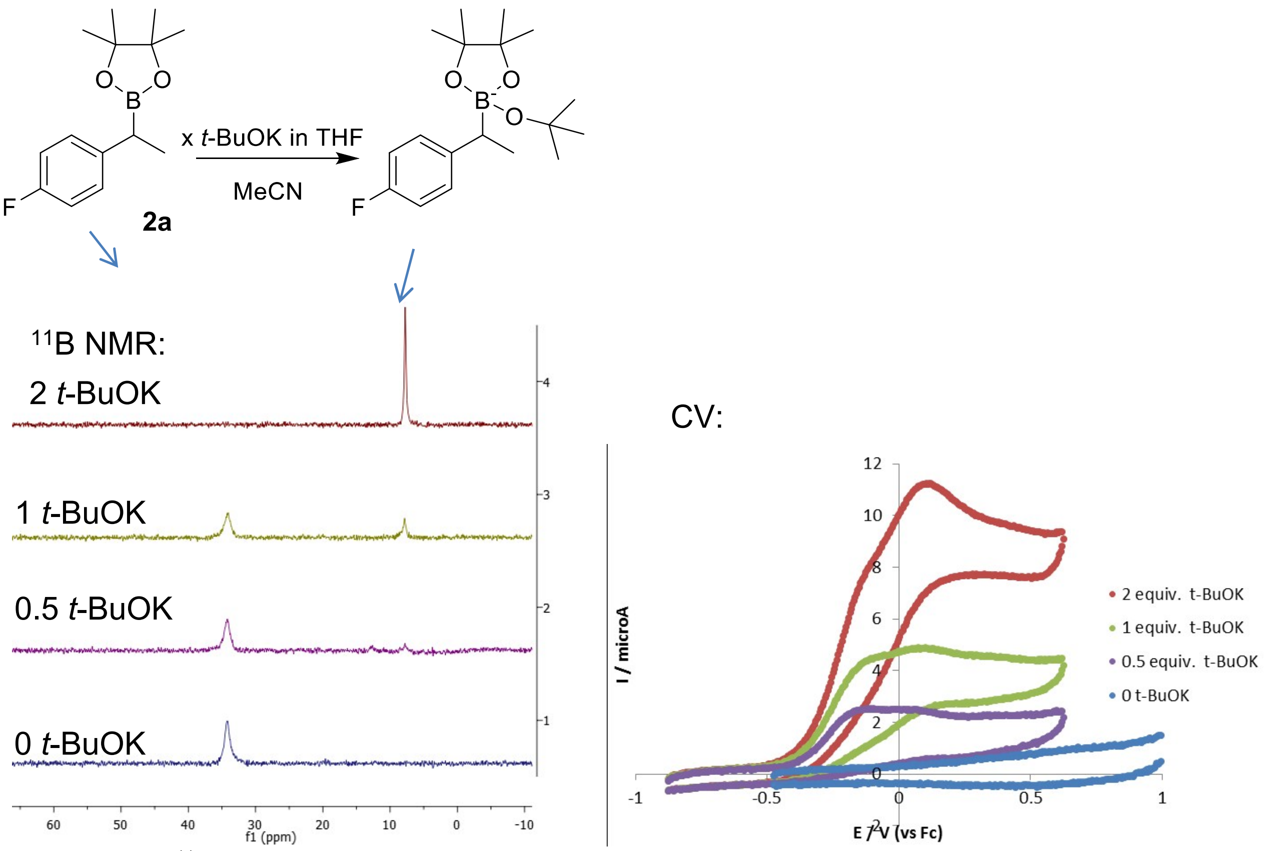

Figure S7:^{11}B NMR of titration of *t*-BuOK into **2a**; Right: CV trace after each addition of *t*-BuOK. **2a** (1 mM), 50 mV/s, WE = GC, Ref = Ag/$AgNO_3$, MeCN, TBAP (100 mM). CV analysis of KO*t*-Bu alone did not reveal any significant current below 0.5 V (vs Fc/Fc^+).

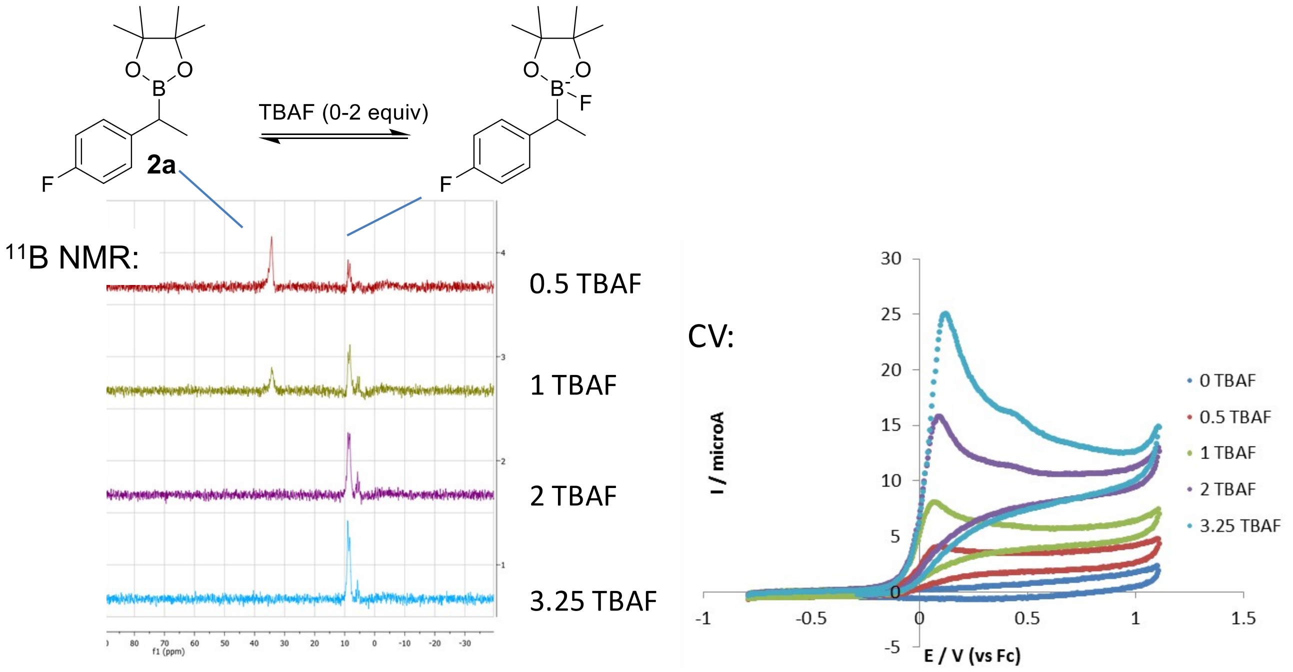

Figure S8:^{11}B NMR of titration of TBAF into **2a**; Right: CV trace after each addition of TBAF. **2a** (1 mM), TBAF (1 M in THF), 50 mV/s, WE = GC, Ref = Ag/$AgNO_3$, MeCN, TBAP (100 mM).

*Oxidation potential study of **1a** with different bases*

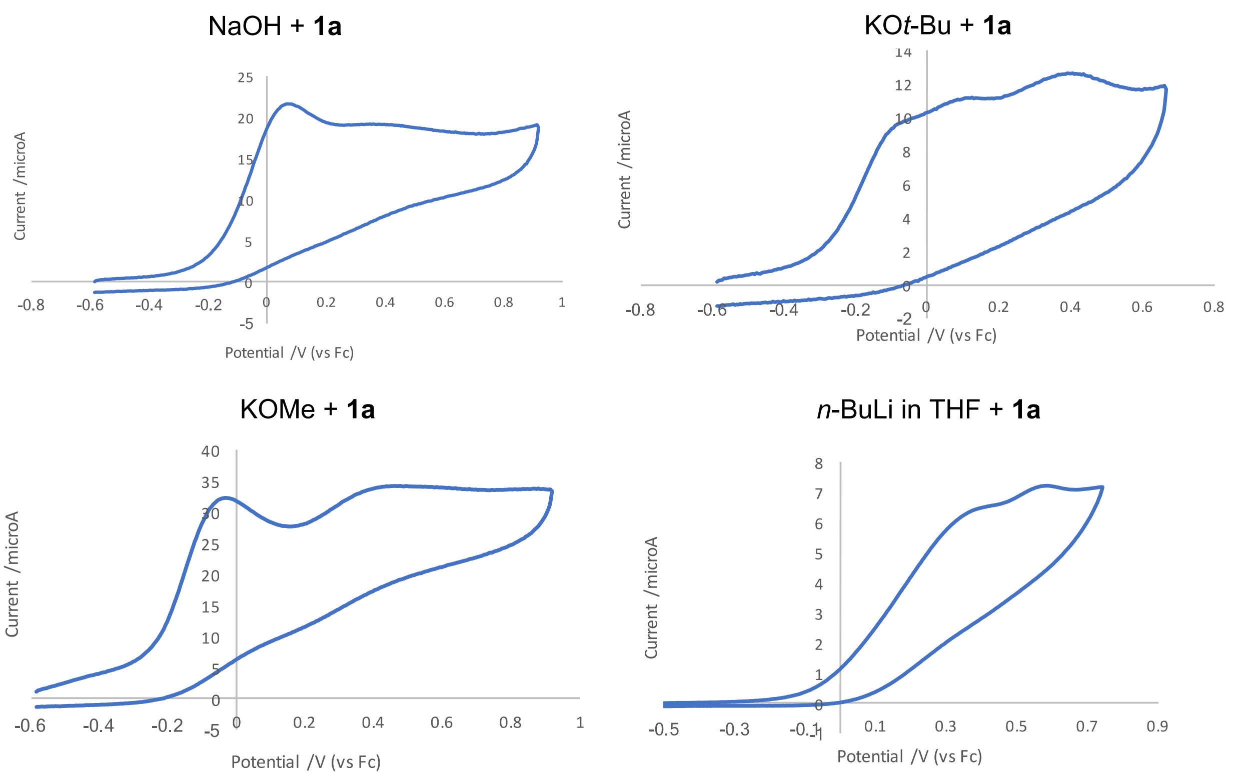

Figure S9: CV traces of boronic ester **1a** with four different bases. **1a** (1 mM), base (2 equiv.), 50 mV/s, WE = GC, Ref = $Ag/AgNO_3$, MeCN, TBAP (100 mM). CV of *n*-BuLi conducted in THF without MeCN. In all cases ^{11}B NMR was used to confirm the formation of the anionic boronate species. For KO*t*-Bu, KOMe and *n*-BuLi, the base was added to a solution of solvent and TBAP containing **1a**. Due to the low solubility of NaOH in MeCN, **1a** and NaOH were premixed in MeCN (1 M) for 20 seconds before being diluted to the bulk solution.

Oxidation potential study of other boronates

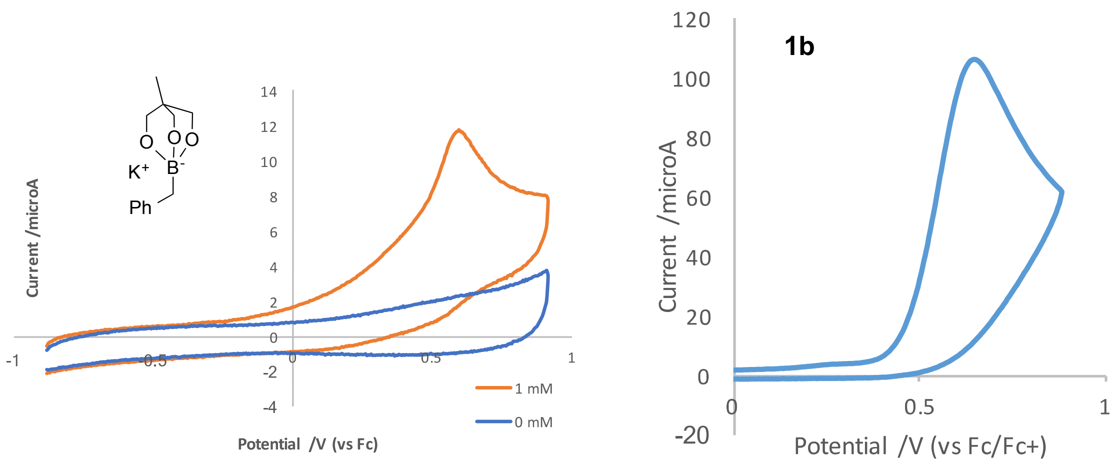

Figure S10: Left: CV trace of cyclic triolboronate (1 mM). Right: **1b** (1 mM), 50 mV/s, WE = GC, Ref = $Ag/AgNO_3$, MeCN, TBAP (100 mM). The cyclic triolboronate was prepared, *vide infra* and **1b** is commercially available.

3. CV studies of electrode fouling

These CV studies were performed by cycling the potential and measuring the current. Following each complete cycle, the solution was stirred and allowed to settle again before initiating another cycle. The electrode was *not* polished in between each cycle. The rate of the electrode fouling was found to be dependent on the concentration of boronate. The current could only be restored upon polishing and not after washing with solvent.

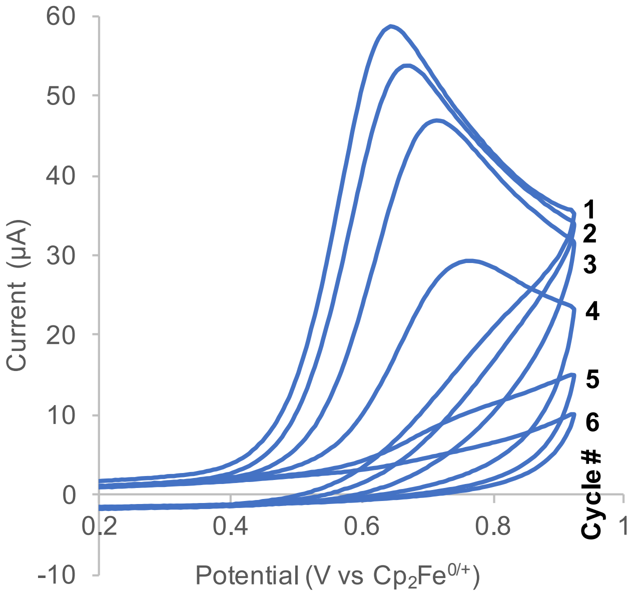

Figure S11: CV of **1b** (0.01 mmol, 2 mM) in MeCN (5 mL) and tetrabutylammonium perchlorate (0.1 M) with a GC disk working electrode (diameter = 3mm). Note, concentration of **1b** in Figure 3 of main text is 5 mM and thus shows a faster rate of electrode fouling than shown in this set of CVs.

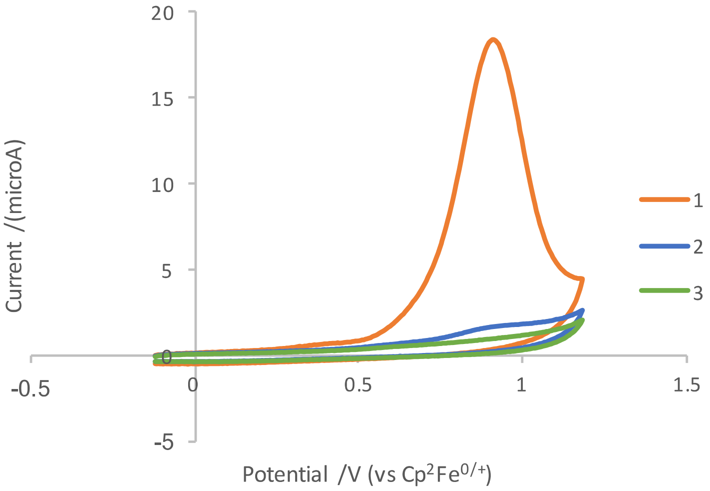

Figure S12: CV of **1b** (0.025 mmol, 5 mM) in MeCN (5 mL) and tetrabutylammonium perchlorate (0.1 M) with a Pt disk working electrode (diameter = 1.6 mm).

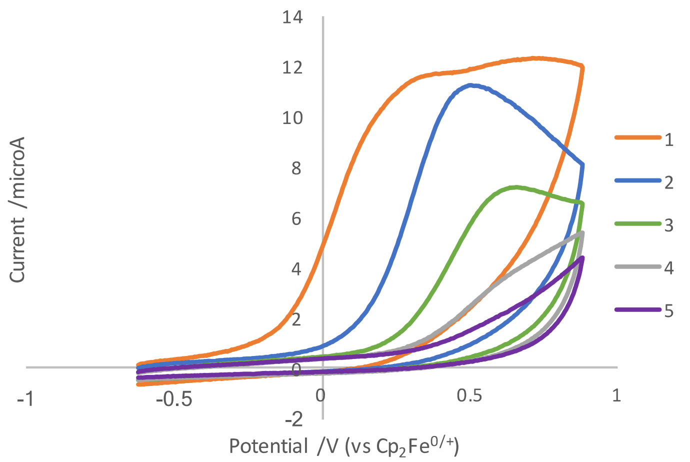

Figure S13: CV of **1a** (0.25 mmol) and NaOH (0.25 mmol) in MeCN (5 mL) and tetrabutylammonium perchlorate (0.1 M) with a Pt disk working electrode (diameter = 1.6 mm). The **1a** and NaOH were premixed in MeCN (1 M) for 20 seconds before being diluted into the bulk solution.

4. SEM analysis of electrode fouling

Electrode preparation for SEM before fouling

A 1 mm diameter glassy carbon (GC) disk electrode was polished, washed with MeCN and dried. A blank CV of only TBAP (0.1 M) in MeCN (5 mL) was run (100 mV/s, -0.5-1.5 V (vs Ag/Ag^+)) followed by a chronoamperometry (CA) experiment (1 V (vs Ag/Ag^+), 60 s) and again a CV (100 mV/s, -0.5-1.5 V (vs Ag/Ag^+)). The surface of the GC electrode was rinsed with fresh MeCN, air-dried, and analysed by SEM (see images below).

Electrode fouling and preparation for SEM

The electrode was polished again, washed with MeCN and dried. The electrode was immersed into a solution of **1b** (50 mM), TBAP (0.1 M) in MeCN (5 mL) and a CV was run (100 mV/s, -0.5-1.5 V (vs Ag/Ag^+)) followed by a chronoamperometry (CA) experiment (1 V (vs Ag/Ag^+), 60 s) and a CV (100 mV/s, -0.5-1.5 V (vs Ag/Ag^+)). These CVs are displayed in Figure S14 and clearly show the fouling effect that has occurred. The surface of the GC electrode was rinsed with fresh MeCN, air-dried, and analysed by SEM (see images below).

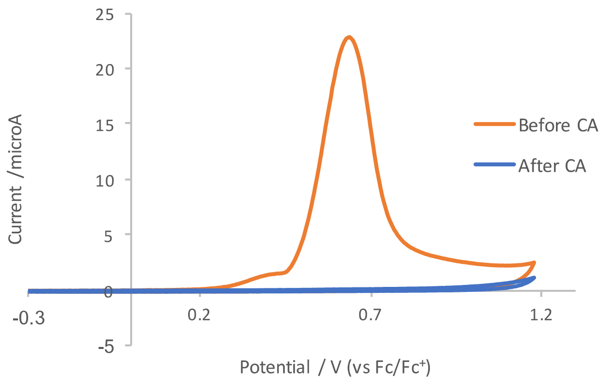

Figure S14: CVs of **1b** in TBAP (0.1 M) in MeCN (5 mL) before and after chronoamperometry (CA) of 1 V for 60 s.

The electrode with the fouled surface was immersed into a solution of Fc (1 mM), TBAP (0.1 mM) in MeCN (5mL) and a CV was run (Figure S15). The electrode was then polished and the CV was run again.

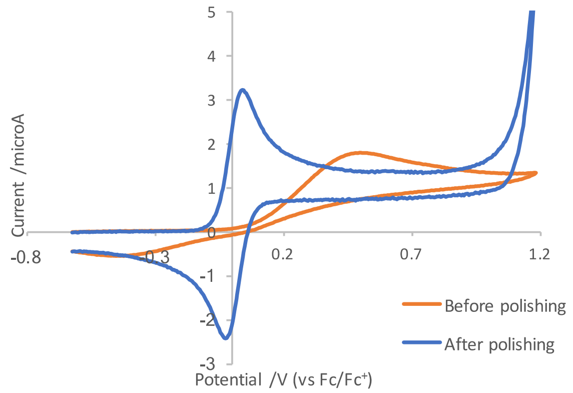

Figure S15: CVs of Fc (1 mM), TBAP (0.1 mM) in MeCN (5mL) using the fouled GC electrode (orange), which is then polished (blue).

SEM images before fouling

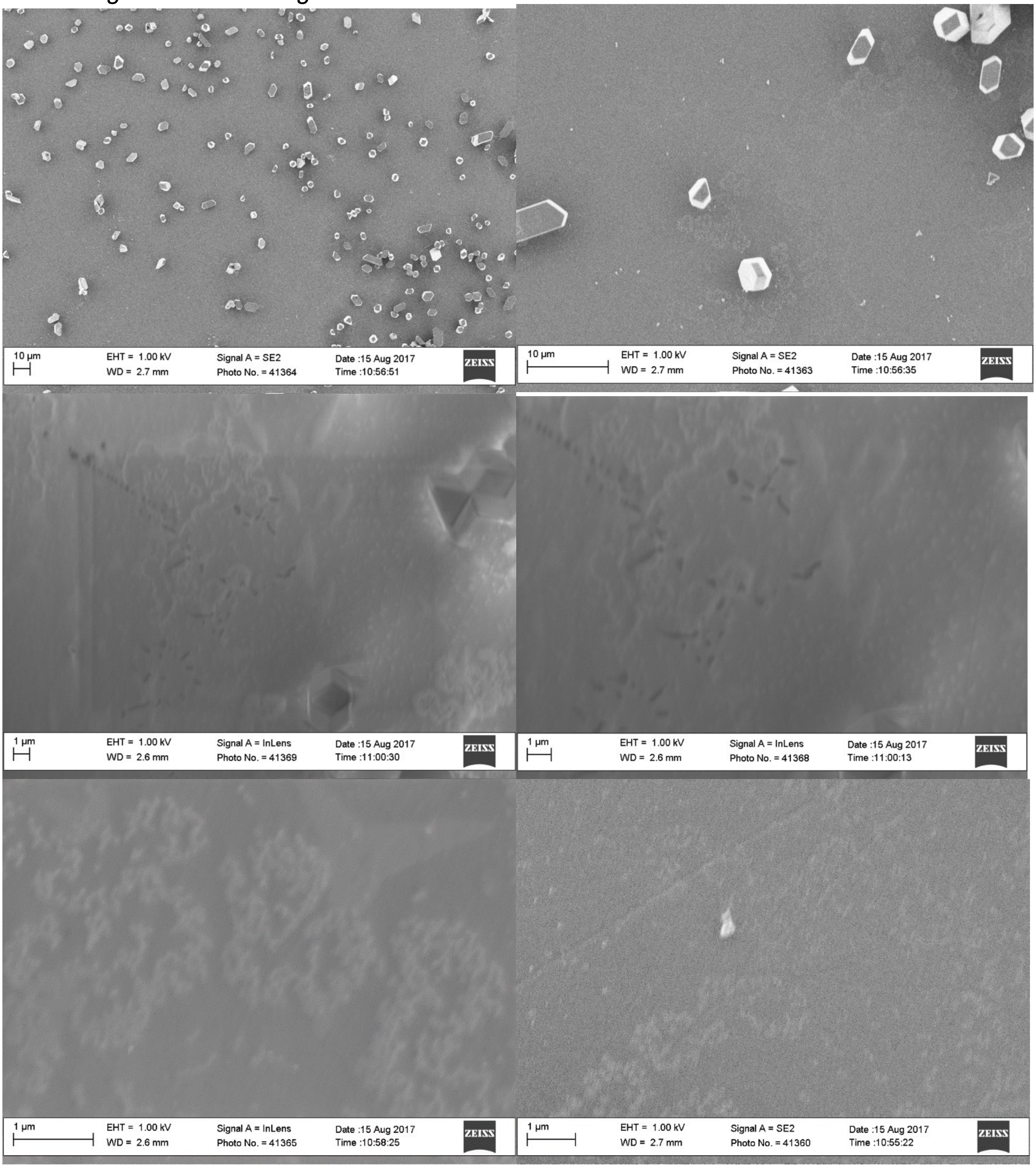

Figure S16: SE2 and InLens images of the GC electrode surface before fouling. Microparticles are likely to come from alumina during the polishing process.

SEM images after fouling

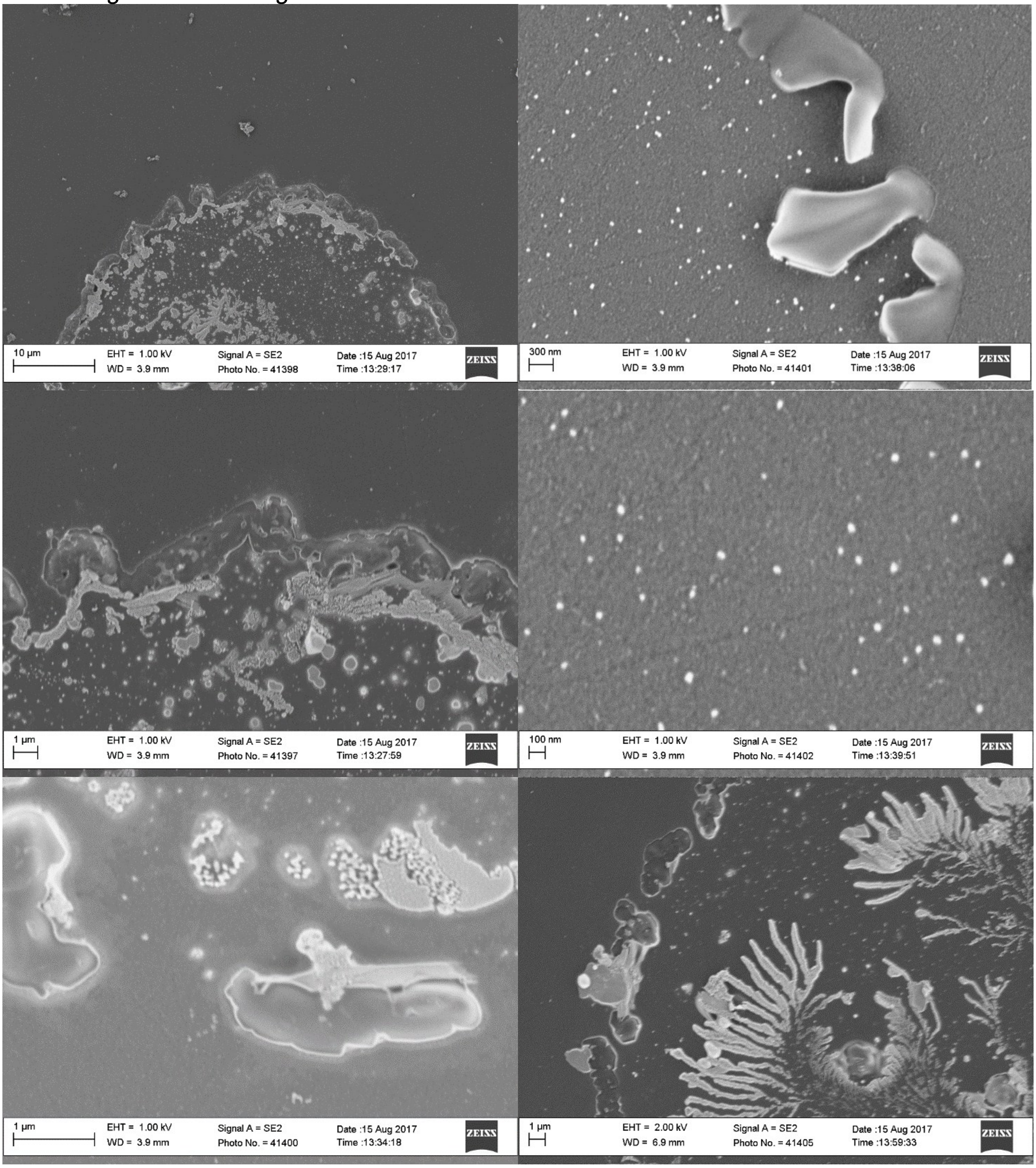

Figure S17: SE2 images of the GC electrode surface after fouling. Amorphous organic deposits found on the surface were only found to cover <10% of the surface, which otherwise appeared relatively clean and free of the microparticles observed before fouling. This suggests a much thinner, molecular covering of the electrode is responsible for the electrical insulation observed by CV and bulk electrolysis.

EDS analysis after fouling

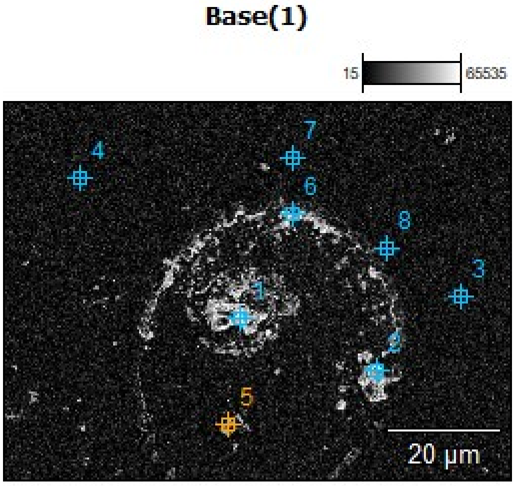

Atom%	C-K		O-K		F-K	
Base(1)_pt1	68.81%	±0.777	18.63%	±1.357	12.57%	±0.748
Base(1)_pt2	64.02%	±0.900	24.56%	±1.537	11.42%	±1.615
Base(1)_pt3	100.00%	±0.590	0.00%	±0.000		
Base(1)_pt4	100.00%	±0.590	0.00%	±0.000		
Base(1)_pt5	100.00%	±0.586	0.00%	±0.000		
Base(1)_pt6	86.88%	±0.661	7.75%	±0.596	5.38%	±0.432
Base(1)_pt7	99.96%	±0.590	0.04%	±0.183		

Figure S18: The amorphous deposits on the electrode surface after fouling were found to contain fluoride originating from **1b**.

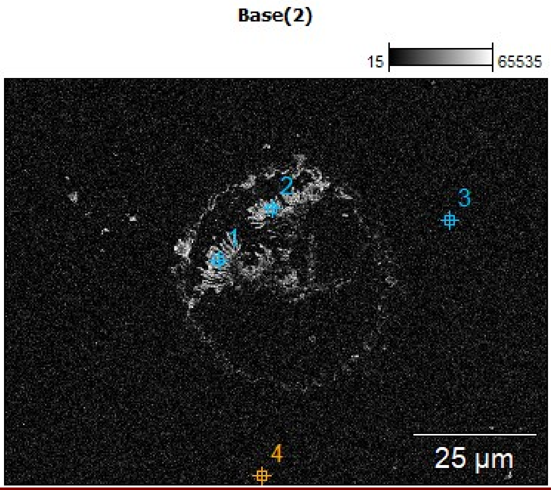

Atom%	C-K	C-K	O-K	O-K	F-K	F-K
Base(2)_pt1	92.59%	±0.833	5.71%	±0.822	1.70%	±0.529
Base(2)_pt2	94.65%	±0.614	3.91%	±0.305	1.44%	±0.327
Base(2)_pt3	100.00%	±0.588	0%	±0.000		
Base(2)_pt4	100.00%	±1.025	0%	±0.000		

Figure S19: The amorphous deposits on the electrode surface after fouling were found to contain fluoride originating from **1b**.

5. CV studies of mediated boronate oxidation

3a *+ NaOH + FcMe*$_8$

3a =

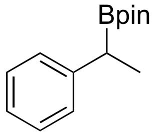

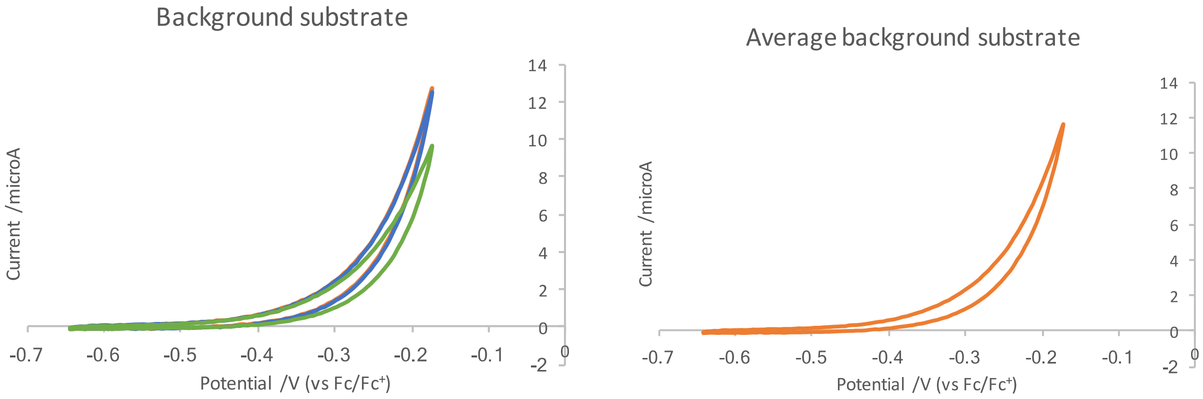

Figure S20: Left: repeated CVs of **3a** (50 mM) with NaOH (50 mM) in MeCN 5 mL, 0.1 M TBAP, 10 mV/s. The glassy carbon electrode was polished in between each cycle. **3a** and NaOH were premixed in MeCN (1 M) for 20 seconds before being diluted into the bulk solution. Right: averaged CV scan of **3a** and NaOH.

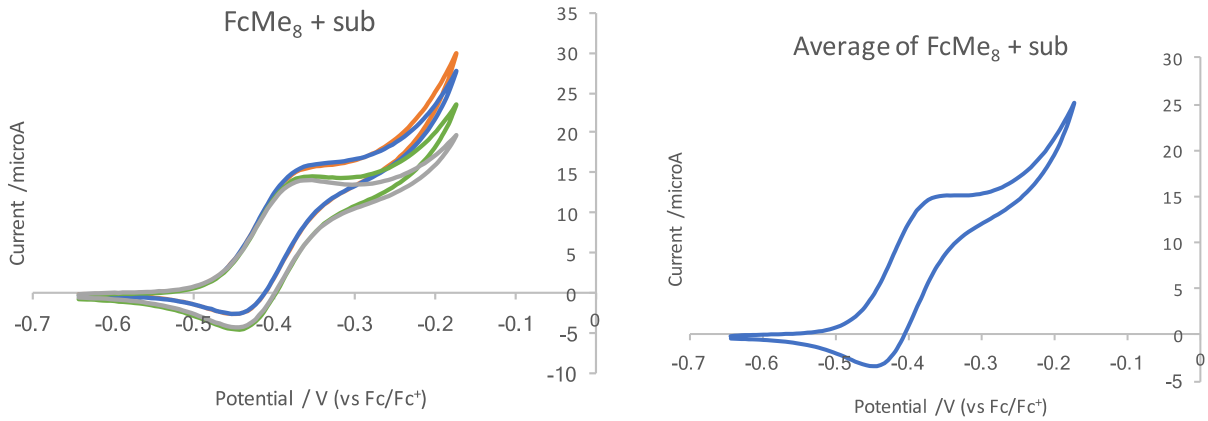

Figure S21: Left: repeated CVs of FcMe$_8$ (1 mM), **3a** (50 mM) with NaOH (50 mM) in MeCN 5 mL, 0.1 M TBAP, 10 mV/s. **3a** and NaOH were premixed in MeCN (1 M) for 20 seconds before being diluted into the bulk solution. The glassy carbon electrode was polished in between each cycle. Right: averaged CV scan of **3a**, NaOH and FcMe$_8$.

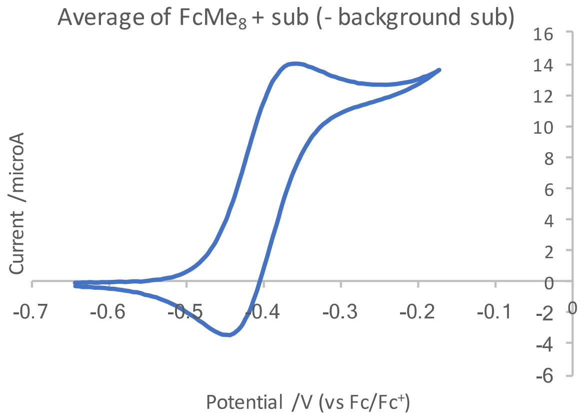

Figure S22: CV resulting from the difference between the averaged **3a**, NaOH + FcMe$_8$ CV (Figure S22) and the averaged background **3a** + NaOH CV (Figure S21).

***1b** + FcBr$_2$*

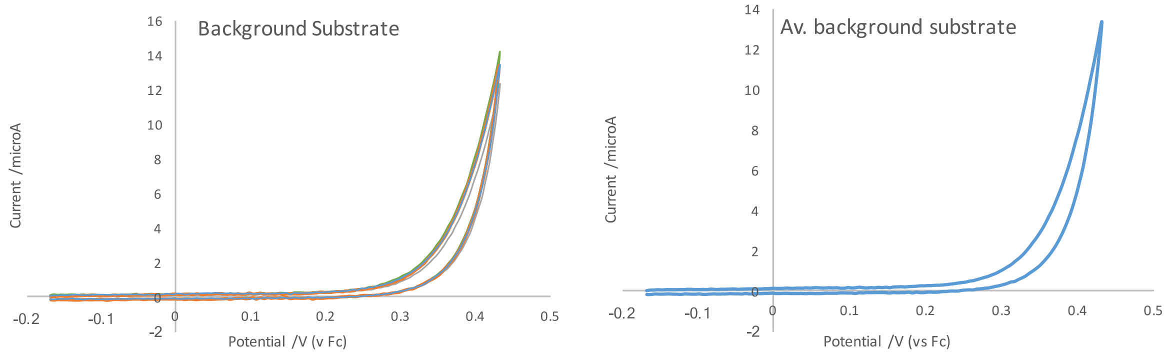

Figure S23: Left: repeated CVs of **1b** (5 mM) in MeCN:THF (1:1) 5 mL, 0.1 M TBAP, 10 mV/s). The glassy carbon electrode was polished in between each cycle. Right: averaged CV scan of **1b**.

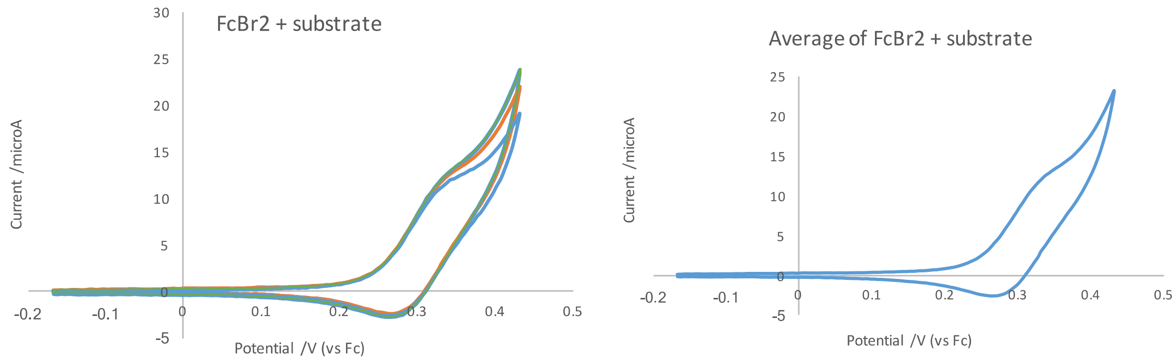

Figure S24: Left: repeated CVs of **1b** (5 mM) and FcBr$_2$ (1 mM) in MeCN:THF (1:1) 5 mL, 0.1 M TBAP, 10 mV/s). The glassy carbon electrode was polished inbetween each cycle. Right: averaged CV scan of **1b** and FcBr$_2$.

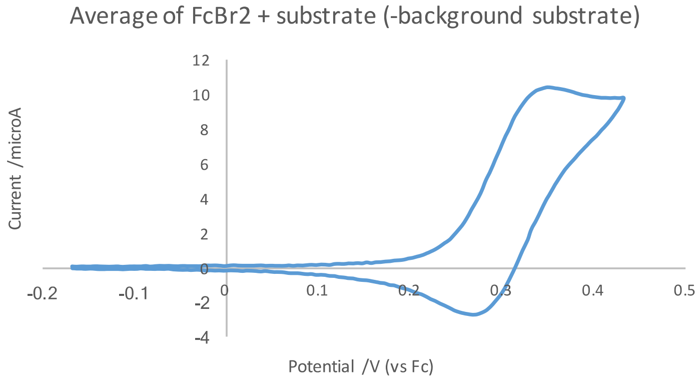

Figure S25: CV resulting from the difference between the averaged **1b** + FcBr$_2$ CV (Figure S24) and the averaged background **1b** CV (Figure S23).

***1b** with other ferrocene mediators*

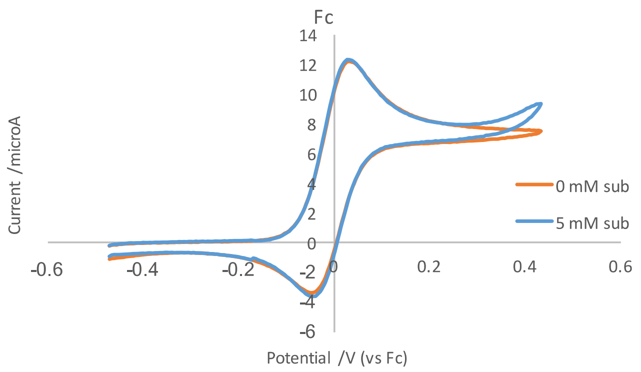

Figure S26: CVs of Fc with and without **1b**. CVs are the average of several runs, with any current due to background direct oxidation of **1b** removed, processed in the same way as shown above for $FcBr_2$.

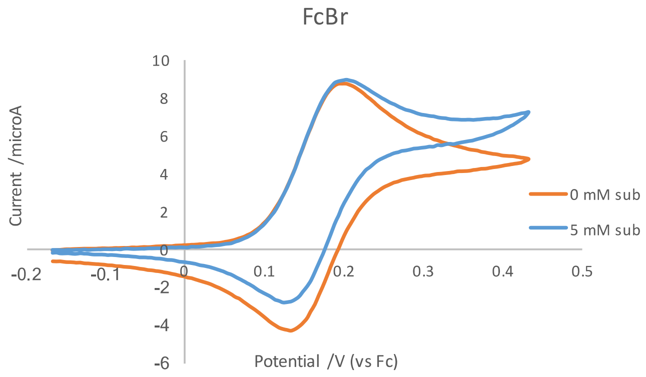

Figure S27: CVs of FcBr with and without substrate **1b**. CVs are the average of several runs, with any current due to background direct oxidation of **1b** removed, processed in the same way as shown above for $FcBr_2$.

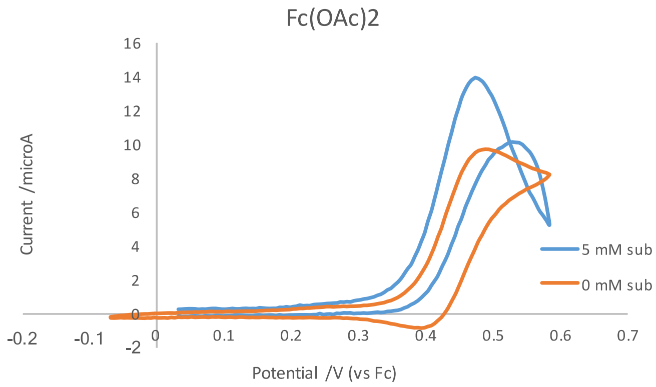

Figure S28: CVs of $Fc(OAc)_2$ with and without substrate **1b**. CVs are the average of several runs, with any current due to background direct oxidation of **1b** removed, processed in the same way as shown above for $FcBr_2$.

6. Bulk electrolyses

General procedure

To the anodic compartment of an oven-dried divided electrochemical cell (Figure S1) was added a magnetic stir bar, Bu_4NClO_4 (171 mg) and an appropriate, catalyst (0.01 mmol). To the cathodic compartment was added Bu_4NClO_4 (171 mg). A reticulated vitreous carbon (RVC) working electrode, $Ag/AgNO_3$ reference electrode, and glassy carbon disk electrode were inserted to the anodic compartment. A platinum wire counter electrode was inserted in the cathodic compartment. The cell was further dried under vacuum and purged with N_2. Under a stream of N_2, TEMPO was added to the anodic compartment. To the anodic and cathodic cells were added 5 mL dry and degassed MeCN.

The benzylic boronic ester **2a** (0.1 mmol) and base (0.2 mmol) were added to the anodic chamber. When NaOH was employed, it was pre-stirred with **2a** in a Teflon-capped 1.5-dram vial charged with a magnetic stir bar that was then purged with N_2 gas. Dry and degassed 0.1-0.2 mL MeCN was added and the solution was stirred vigorously for 30 s. The solution was then transferred to the anodic compartment of the electrochemical cell.

A CV was obtained of the solution using the glassy carbon disk electrode. If running a constant potential experiment, the potential chosen to be applied was 50-100 mV lower than the relevant oxidation peak. If running a constant current experiment, a 20 s chronoamperogram was collected with a potential applied at 30 mV lower than the relevant peak potential. Bulk electrolysis was then carried out at half the current measured with this chronoamperogram. The reaction was stopped after 1.5 F/mol had been applied or after the measured potential of the solution increased to the oxidation potential of excess TEMPO. A sample from the reaction was then removed and analysed by ^{19}F NMR.

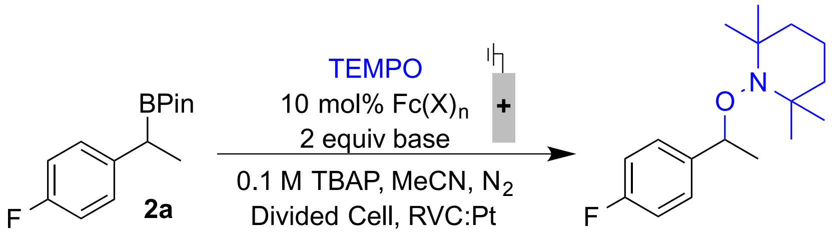

Entry	TEMPO equiv.	Base	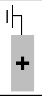	Catalyst	Catholyte Additive	MB (%)	Yield (%)[a]
1	4	TBAF	0.0 V	No Catalyst	-	60	47
2	4	NaOH[b]	0.4 mA	No Catalyst	-	76	65
3	4	TBAF	0.0 V	$Fc(Me)_2$	-	85	31
4	4	KO*t*Bu	-0.4 V	$Fc(Me)_8$	-	103	69
5	1.5	NaOH	-0.4 V	$Fc(Me)_8$	-	87	44
6	1.5	NaOH[b]	-0.4 V	$Fc(Me)_8$	-	85	71
7	1.5	NaOH[b]	-0.4 V	$Fc(Me)_8$	H_2O	85	85
8	1.5	NaOH[b]	0.4 mA	$Fc(Me)_8$	H_2O	89	85
9[c]	1.5	NaOH[b]	0.4 mA	No Catalyst	H_2O	89	16
10	1.5	NaOH[b]	0.4 mA	$Fc(Me)_8$	AcOH	100	99

[a] ^{19}F NMR yield with 4,4'-difluorobiphenyl internal standard, 0.1 mmol (10 mM) of **2a**

[b] 25 additional equivalents of powdered NaOH added into the bulk solution

[c] Pt mesh electrode used as anode

Entries 1 and 2 demonstrate the direct oxidation of **2a** with two different bases and trapping with a large excess of TEMPO. Introducing a catalyst to the system (Entry 3) did not improve the yield when TBAF was used as a base due to deactivating interactions between ferrocenium and fluoride, see reference 26 in main text. Employing the strong base, sodium *tert*-butoxide provided improved yields and mass balance (Entry 4). The butoxide anion did not show deactivating interactions with the electrochemically generated ferrocenium derivative. NaOH, which is sufficiently insoluble in MeCN, provided an acceptable mass balance without catalyst deactivation (Entry 5). An increased

loading of NaOH (Entry 6) in the bulk solution increased the NMR yield of product, presumably by helping to retain formation of the anionic boronate in solution. A variety of proton sources were tested to facilitate proton reduction at the counter electrode. Strong acids are commonly added[4–9] as sacrificial oxidants, but we found that water led to good increases in yield (Entry 7). Running the reaction with constant current led to an identical reaction outcome (Entry 8). Analysis of the applied potential under constant current conditions, was found to remain more consistent over the duration of the reaction, if a proton source (sacrificial oxidant) was added. Acetic acid as sacrificial oxidant was also found to facilitate catalysis and yielded the highest yield and mass balance observed (Entry 10). Finally, running the reaction with a platinum working electrode in place of the RVC one, resulted in a very poor yield (Entry 9).

7. Reactions with stoichiometric oxidant

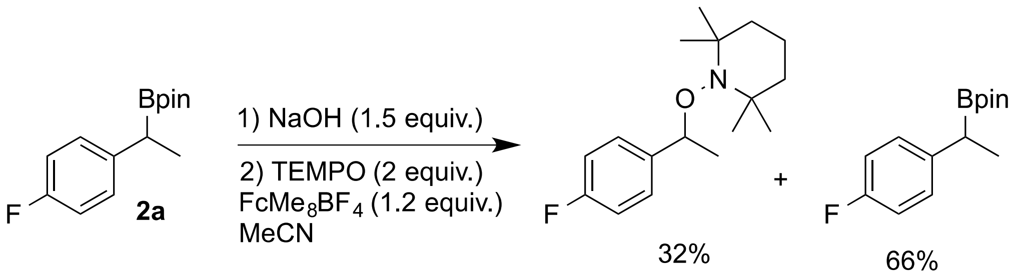

2a (0.05 mmol) and NaOH (0.075 mmol) were added to a N_2 purged vial and stirred with MeCN (1 M) for 30 seconds. This was then diluted into a solution containing TEMPO (0.1 mmol) and 4,4'-difluorobiphenyl (0.01 mmol) in MeCN (0.5 mL). To this was added dropwise a solution containing $FcMe_8BF_4$ (0.024 mmol) in MeCN (0.5 mL) and stirred for 10 min before being analysed by ^{19}F NMR.

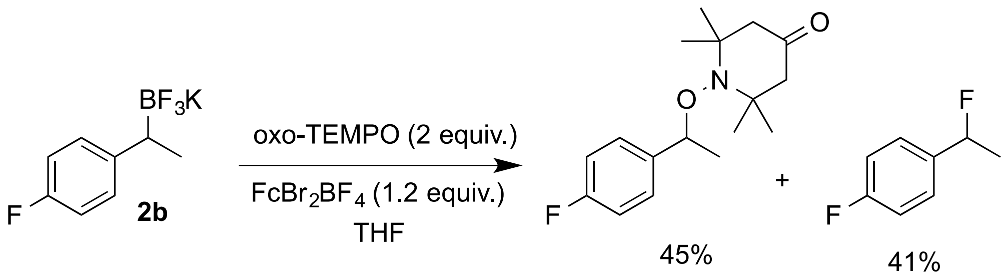

A solution containing $FcBr_2BF_4$ (0.024 mmol) in THF (0.5 mL) was added dropwise to a solution of **2b** (0.02 mmol), 4,4'-difluorobiphenyl (0.005 mmol) and oxo-TEMPO (0.04 mmol) in THF (0.5 mL) with stirring for 10 min before being analysed by ^{19}F NMR. Oxo-TEMPO was used in this reaction for its enhanced oxidative stability compared to TEMPO, which is necessary with the more highly oxidising ferrocenium oxidant.

8. Synthesis and characterisation of TEMPO-trapped products

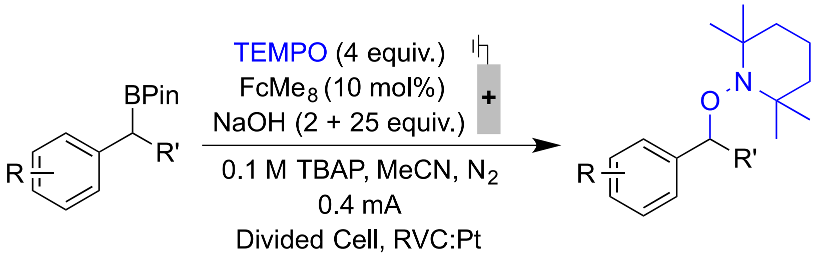

To the anodic compartment of an oven-dried divided electrochemical cell (Figure S2) was added a magnetic stir bar, $Fc(Me)_8$ (3 mg, 0.01 mmol), powdered NaOH (50 mg), and Bu_4NClO_4 (171 mg). To the cathodic compartment was added Bu_4NClO_4 (20 mg). A reticulated vitreous carbon (RVC) working electrode, $Ag/AgNO_3$ reference electrode, and glassy carbon disk electrode were inserted to the anodic compartment. A platinum wire counter electrode was inserted in the cathodic compartment. The cell was further dried under vacuum and purged with N_2. Under a stream of N_2, TEMPO (64 mg, 0.4 mmol) was added to the anodic compartment. To the anodic cell was added 5 mL dry and degassed MeCN. To the cathodic cell was added 0.1 mL degassed H_2O and an appropriate volume of dry and degassed MeCN.

The benzylic boronic ester (0.1 mmol) and powdered NaOH (7 mg, 0.2 mmol) were added to a Teflon-capped 1.5-dram vial charged with a magnetic stir bar that was then purged with N_2 gas. Dry and degassed 0.2 mL MeCN was added to the vial and the solution was stirred vigorously for 30 s. The solution was then transferred to the anodic compartment of the electrochemical cell. Methyl benzoate was added to the anodic compartment as an internal standard. A CV was obtained of the solution using the glassy carbon electrode, and the peak potential of $Fc(Me)_8$ was established. Bulk electrolysis was carried out at 0.4 mA. The electrolysis was stopped after 1.5 F/mol had been applied or after the measured potential of the solution began to increase, whichever came first. After the electrolysis, an aliquot was taken up from the anodic compartment under N_2 for subsequent NMR analysis.

Reactions were also performed on 0.5 mmol, 0.1 M scale relative to the benzylic boronic ester for product isolation. After electrolysis, the reaction solution was concentrated *in vacuo*, triturated with diethyl ether (3 x 10 mL) and passed through a short plug of silica with 50 mL diethyl ether. The crude solution was concentrated *in vacuo* and purified by flash column chromatography. The chromatography conditions are specified for each product.

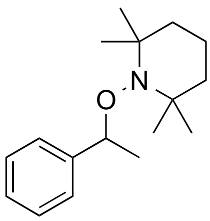

2,2,6,6-tetramethyl-1-(1-phenylethoxy)piperidine.[10] Chromatography with pentane to 10% ethyl acetate in pentane to yield a yellow oil. **^{1}H NMR** (500 MHz, Chloroform-*d*) δ 7.34 – 7.18 (m, 5H), 4.77 (q, *J* = 6.7 Hz, 1H), 1.51-1.28 (m, 6 h), 1.48 (d, *J* = 6.7 Hz, 4H), 1.29 (s, 3H), 1.16 (s, 3H), 1.02 (s, 3H), 0.65 (s, 3H). **^{13}C NMR** (126 MHz, $CDCl_3$) δ 145.98, 128.12, 126.89, 126.73, 83.26, 59.80, 40.51, 34.61, 34.27, 23.72, 20.48, 17.38. **HRMS (ESI)** exact mass calculated for $[M+H]^+$ ($C_{17}H_{28}NO$) requires m/z 262.2165, found m/z 262.2163, difference 0.4 ppm. $Fc(Me)_8$ is detected in the mass spec, but compound is pure by NMR analysis.

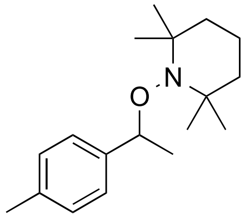

2,2,6,6-tetramethyl-1-(1-(p-tolyl)ethoxy)piperidine.[10] Chromatography with pentane to 5% ethyl acetate in pentane to yield a white solid. **^{1}H NMR** (500 MHz, Chloroform-*d*) δ 7.21 (dd, *J* = 8.1, 2.0 Hz, 2H), 7.11 (d, *J* = 7.6 Hz, 2H), 4.75 (q, *J* = 6.7 Hz, 1H), 2.33 (s, 3H), 1.51-1.28 (s, 6 H), 1.46 (d, *J* = 6.7 Hz, 3H), 1.29 (s, 3H), 1.16 (s, 3H), 1.03 (s, 3H), 0.69 (s, 3H). **^{13}C NMR** (126 MHz, $CDCl_3$) δ 143.00, 136.38, 128.80, 126.64, 82.97, 59.79, 40.51, 34.59, 34.37, 23.74, 21.30, 20.49, 17.39. **HRMS (ESI)** exact mass calculated for $[M+H]^+$ ($C_{18}H_{30}NO$) requires m/z 276.2322, found m/z 276.2321, difference 0.4 ppm.

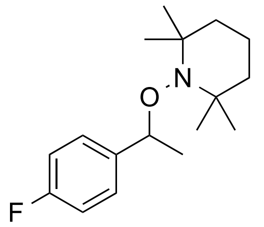

1-(1-(4-fluorophenyl)ethoxy)-2,2,6,6-tetramethylpiperidine.[10] Chromatography with pentane to 10% ethyl acetate in pentane to yield a white solid. **^{1}H NMR** (400 MHz, Chloroform-*d*) δ 7.27 (dd, *J* = 8.6, 5.6 Hz, 2H), 6.99 (t, *J* = 8.8 Hz, 1H), 4.76 (q, *J* = 6.7 Hz, 1H), 1.51-1.28 (m, 6H), 1.46 (d, *J* = 6.7 Hz, 3H), 1.28 (s, 3H), 1.15 (s, 3H), 1.01 (s, 3H), 0.62 (s, 3H). **^{13}C NMR** (126 MHz, $CDCl_3$) δ 161.94 (d, *J* = 244.2 Hz), 141.71, 141.68, 128.34, 128.28, 114.97, 114.80, 82.51, 59.95, 59.72, 40.48, 34.56, 34.35, 23.57, 20.46, 17.35. **HRMS (ESI)** exact mass calculated for $[M+NH_4]^+$ ($C_{17}H_{30}FN_2O$) requires m/z 280.2071, found m/z 280.2072, difference 0.4 ppm.

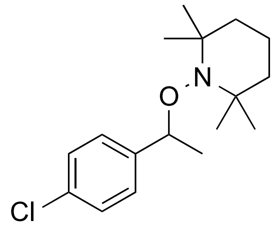

1-(1-(4-chlorophenyl)ethoxy)-2,2,6,6-tetramethylpiperidine.[10] Chromatography with pentane to 5% ethyl acetate in pentane to yield a white solid. **^{1}H NMR** (400 MHz, Chloroform-*d*) δ 7.32 – 7.20 (m, 4H), 4.75 (q, *J* = 6.7 Hz, 1H), 1.28-1.51 (m, 6 H), 1.45 (d, *J* = 6.7 Hz, 2H), 1.28 (s, 3H), 1.15 (s, 3H), 1.01 (s, 3H), 0.64 (s, 3H). **^{13}C NMR** (101 MHz, $CDCl_3$) δ 144.46, 132.50, 128.30, 128.11, 82.62, 59.80 (br), 40.48, 34.58, 34.45, 23.64, 20.46, 17.34. **HRMS (ESI)** exact mass calculated for $[M+H]^+$ ($C_{17}H_{27}ClNO$) requires m/z 296.1776, found m/z 296.1775, difference 0.3 ppm. $Fc(Me)_8$ is detected in the mass spec, but compound is pure by NMR analysis.

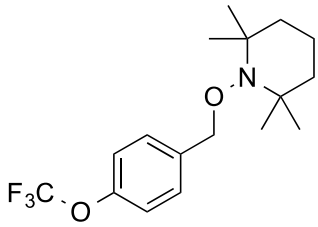

1-(1-(4-trifluoromethyoxyphenyl)mzethoxy)-2,2,6,6-tetramethylpiperidine.[10] Electrolysis performed with $Fc(Me)_2$ (0.01 mmol, 2.1 mg) rather than $Fc(Me)_8$. Chromatography with pentane to 5% ethyl acetate in pentane to yield a white solid. **^{1}H NMR** (400 MHz, Chloroform-*d*) δ 7.38 (d, *J* = 8.6 Hz, 2H), 7.18 (d, *J* = 8.1 Hz, 2H), 4.82 (s, 2H), 1.66 – 1.30 (m, 6H), 1.24 (s, 3H), 1.15 (s, 3H). **^{13}C NMR** (126 MHz, Chloroform-*d*) δ 148.50 (q, *J* = 1.8 Hz), 137.21, 128.75, 120.93, 120.66 (q, *J* = 256.8 Hz), 77.96, 60.21, 39.87, 33.22, 20.44, 17.24. **HRMS (ESI)** exact mass calculated for $[M+H]^+$ ($C_{17}H_{25}F_3NO_2$) requires m/z 332.1832, found m/z 332.1829, difference 0.9 ppm.

9. Synthesis of benzylic boron compounds

*Benzylic neopentylglycol boronic ester (**1c**)*

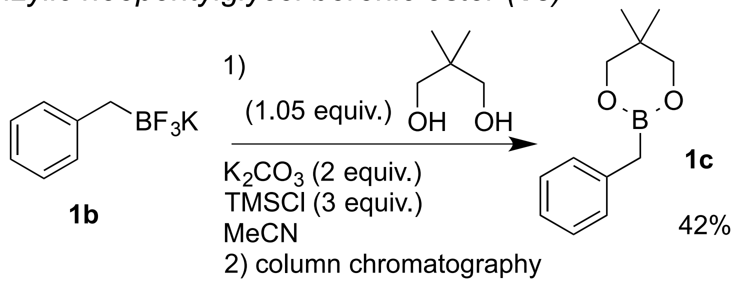

1c was prepared on a 0.2 mmol scale according to a literature method[2] (17 mg, 42%). **^{1}H NMR** (400 MHz, Chloroform-*d*) δ 7.27 – 7.20 (m, 2H), 7.19 – 7.14 (m, 2H), 7.15 – 7.02 (m, 1H), 3.58 (s, 4H), 2.22 (s, 2H), 0.92 (s, 6H); **^{11}B NMR** (128 MHz, Chloroform-*d*) δ 29.37.

*Benzylic diaminoboronamide (**1d**)*

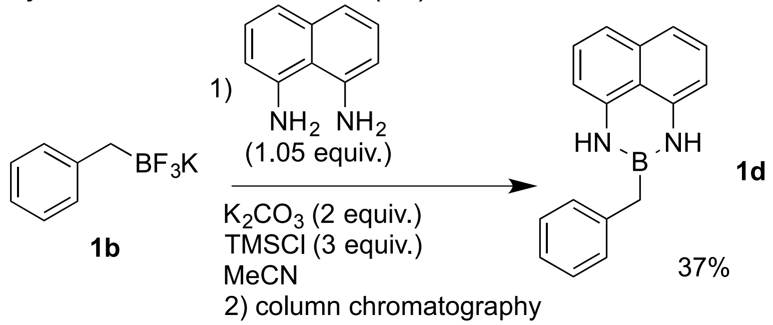

1d was prepared on a 0.2 mmol scale according to a literature method[2] that gave **1d** as an orange solid (19 mg, 37%). **^{1}H NMR** (400 MHz, Chloroform-*d*) δ 7.31 (t, *J* = 7.5 Hz, 2H), 7.22 – 7.11 (m, 3H), 7.10 – 7.03 (m, 2H), 6.99 (dd, *J* = 8.3, 1.1 Hz, 2H), 6.22 (dd, *J* = 7.2, 1.1 Hz, 2H), 5.47 (s, 2H), 2.38 (s, 2H); **^{11}B NMR** (128 MHz, Chloroform-*d*) δ 31.42.

Potassium benzylic cyclic triolboronate

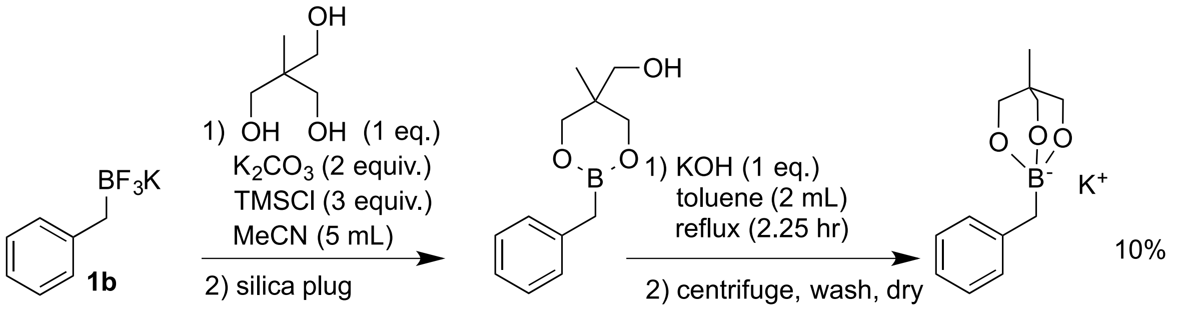

TMSCl (3 equiv., 1.2 mmol, 129 mg) was added to a solution of potassium benzyltrifluoroborate **1b** (1 equiv., 0.4 mmol, 79 mg), 2-(hydroxymethyl)-2-methylpropane-1,3-diol (1 equiv., 0.4 mmol, 48 mg), K_2CO_3 (2 equiv., 0.8 mmol, 110 mg), in MeCN (5 mL) and stirred for 2 hours. The solution was taken through a pad of silica gel, eluting with EtOAc (20 mL) and concentrated. Toluene (2 mL) and KOH (1 equiv., 0.4 mmol, 22.4 mg) were then added and heated to reflux for 2 hours. The solution was allowed to cool before the solid was collected by centrifugation and washed with additional aliquots of toluene (3 x 5 mL). The solid was dissolved into MeCN (2 mL) and taken through a 0.4 μm Whatman syringe filter to yield a white solid (10 mg, 10% yield).

^{11}B NMR (128 MHz, DMF-d$_7$) δ 4.06; **^{1}H NMR** (400 MHz, DMF-*d*$_7$) δ 7.10 (d, *J* = 7.3 Hz, 2H), 6.95 (t, *J* = 7.5 Hz, 1H), 6.78 (t, *J* = 7.2 Hz, 1H), 3.58 (s, 2H), 1.57 (s, 1H), 0.42 (s, 1H); ^{13}C NMR (101 MHz, MeCN-*d*$_3$) δ 129.42 (s), 128.61 (s), 124.96 (s), 66.67 (s), 17.05 (s) <u>C</u>H-B not observed

10. Synthesis of pinacol benzylic boronic esters

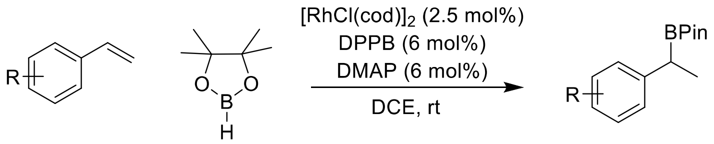

Pinacol boronic esters were synthesized according to a literature procedure.[1] To a solution of $[RhCl(cod)_2]_2$ (49.4 mg, 0.1 mmol), 1,4-bis(diphenylphosphino)butane (DPPB) (102.4 mg, 0.24 mmol), and 4-dimethylaminopyridine (DMAP) (29.4 mg, 0.24 mmol) in 4 mL of 1,2-dichloromethane, was added pinacolborane (0.68 mL, 4.8 mmol) and styrene derivative (4 mmol). The reaction mixture was stirred under N_2 for two hours and filtered through a silica plug with diethyl ether (50 mL). The crude mixture was concentrated *in vacuo* and purified by column chromatography. All accessed products are known compounds and analytical data matched the results from literature.

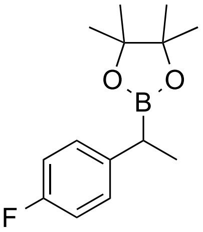

2-(1-(4-fluorophenyl)ethyl)-4,4,5,5-tetramethyl-1,3,2-dioxaborolane ***(2a)***.[11] Synthesized according to general procedure. Chromatography with pentane to 5% ethyl acetate in pentane. Colourless oil obtained in 80% yield. **^{1}H NMR** (500 MHz, Chloroform-*d*) δ 7.20 – 7.12 (m, 2H), 6.99 – 6.89 (m, 2H), 2.41 (q, *J* = 7.5 Hz, 1H), 1.31 (d, *J* = 7.5 Hz, 3H), 1.20 (d, *J* = 5.8 Hz, 12H). **^{13}C NMR** (126 MHz, $CDCl_3$) δ 161.96, 160.03, 140.63, 129.10, 115.18, 115.02, 83.50, 77.16, 24.76, 24.72, 17.36. **^{11}B NMR** (128 MHz, $CDCl_3$) δ 33.48. **^{19}F NMR** (377 MHz, $CDCl_3$) δ -119.06. *C*H-BPin not observed.

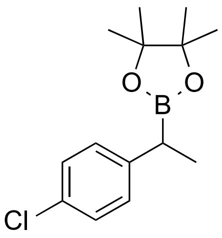

2-(1-(4-chlorophenyl)ethyl)-4,4,5,5-tetramethyl-1,3,2-dioxaborolane.[11] Synthesized according to general procedure. Chromatography with pentane to 5% ethyl acetate in pentane. Colourless oil obtained in 73% yield. **^{1}H NMR** (400 MHz, Chloroform-*d*) δ 7.24 – 7.20 (m, 1H), 7.17 – 7.12 (m, 1H), 2.40 (q, *J* = 7.5 Hz, 1H), 1.30 (d, *J* = 7.5 Hz, 2H), 1.20 (d, *J* = 4.2 Hz, 6H). **^{13}C NMR** (126 MHz, $CDCl_3$) δ 143.87, 131.12, 129.48, 128.74, 83.82, 77.16, 25.02, 24.98, 17.33. **^{11}B NMR** (128 MHz, $CDCl_3$) δ 33.39. *C*H-BPin not observed.

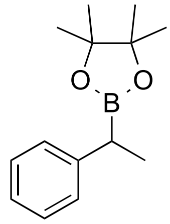

2-(1-phenyl-ethyl)-4,4,5,5-tetramethyl-1,3,2-dioxaborolane.[11] Synthesized according to general procedure. Chromatography with pentane to 5% ethyl acetate in pentane. Colourless oil obtained in 49% yield. **^{1}H NMR** (400 MHz, Chloroform-*d*) δ 7.31 – 7.07 (m, 5H), 2.43 (q, *J* = 7.5 Hz, 1H), 1.33 (dd, *J* = 7.5, 0.9 Hz, 3H), 1.20 (d, *J* = 5.5 Hz, 12H). **^{13}C NMR** (126 MHz, $CDCl_3$) δ 145.32, 128.65, 128.14, 125.43, 83.63, 77.16, 25.00, 24.96, 17.43. **^{11}B NMR** (128 MHz, $CDCl_3$) δ 33.58. *C*H-BPin not observed.

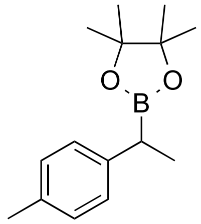

2-(1-(4-methylphenyl)ethyl)-4,4,5,5-tetramethyl-1,3,2-dioxaborolane.[11] Synthesized according to general procedure. Chromatography with hexane to 80% ethyl acetate in hexane. Colourless oil obtained in 81% yield. **^{1}H NMR** (400 MHz, Chloroform-*d*) δ 7.14 (d, *J* = 8.1 Hz, 2H), 7.09 (d, *J* = 8.0 Hz, 2H), 2.41 (q, *J* = 7.5 Hz, 1H), 2.32 (s, 3H), 1.33 (d, *J* = 7.5 Hz, 3H), 1.23 (d, *J* = 5.1 Hz, 12H). **^{13}C NMR** (101 MHz, Chloroform-*d*) δ 141.99, 134.48, 129.14, 127.77, 83.33, 24.75, 24.72, 21.09, 17.42. *C*H-BPin not observed.

11. Spectra

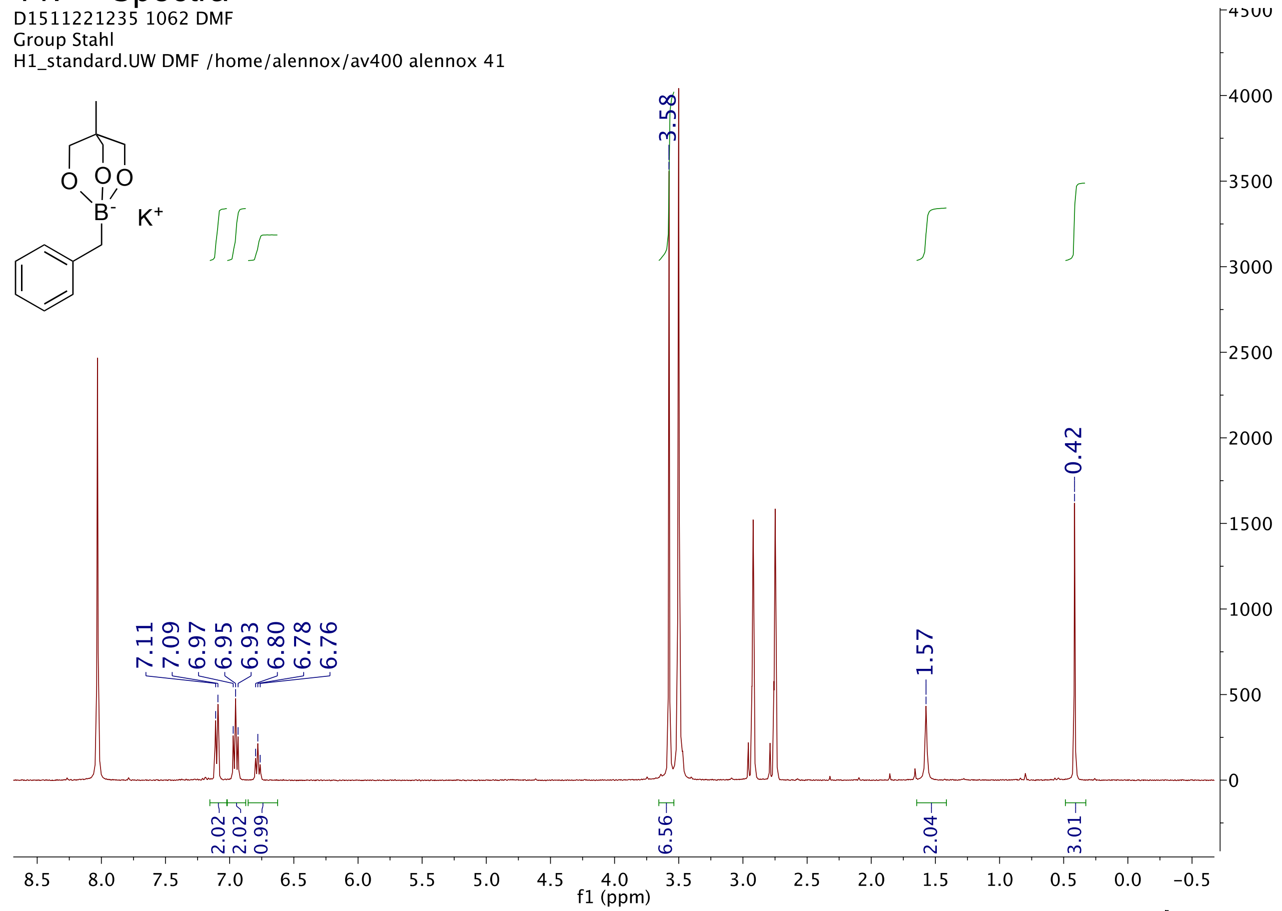
D1511221235 1062 DMF
Group Stahl
H1_standard.UW DMF /home/alennox/av400 alennox 41
K+
7.11
7.09
6.97
6.95
6.93
6.80
6.78
6.76
3.58
1.57
0.42
2.02
2.02
0.99
6.56
2.04
3.01
f1 (ppm)

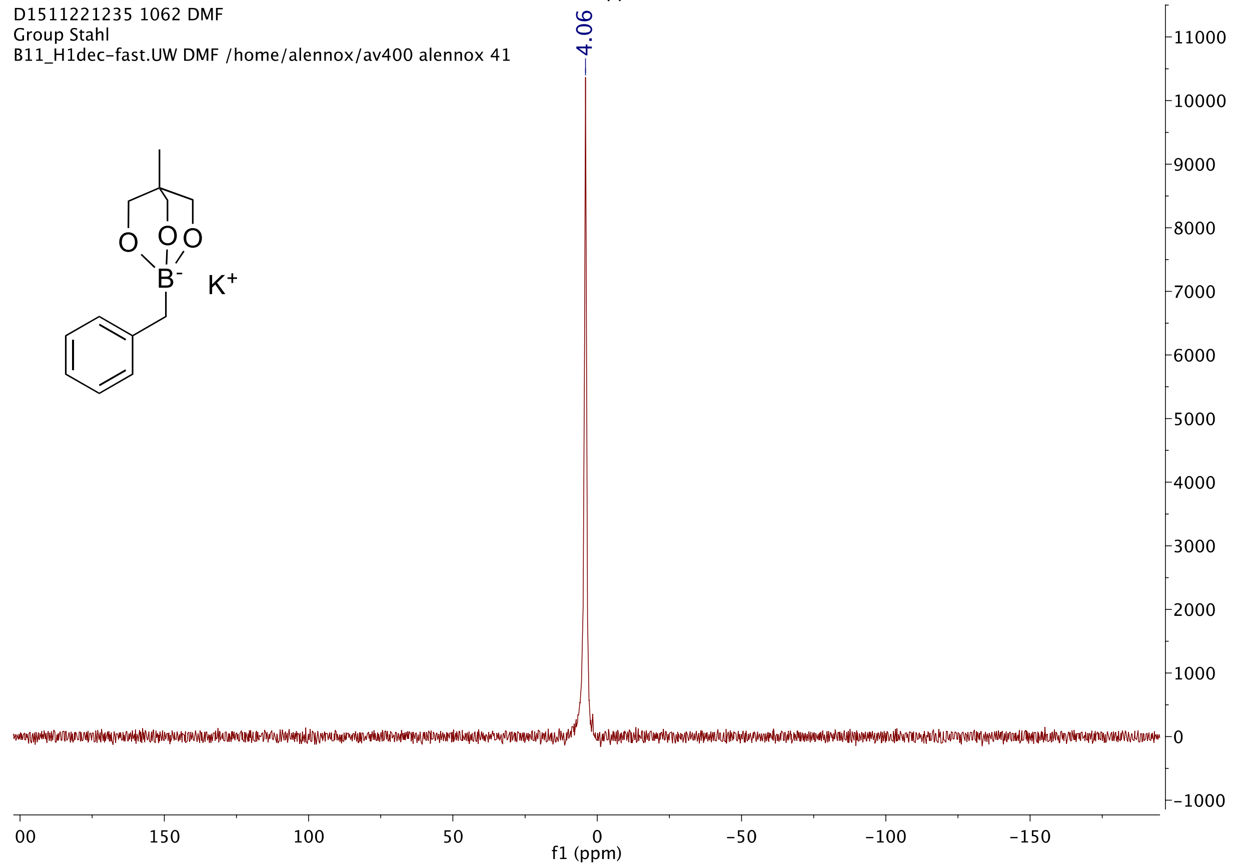
D1511221235 1062 DMF
Group Stahl
B11_H1dec-fast.UW DMF /home/alennox/av400 alennox 41
K+
4.06
f1 (ppm)

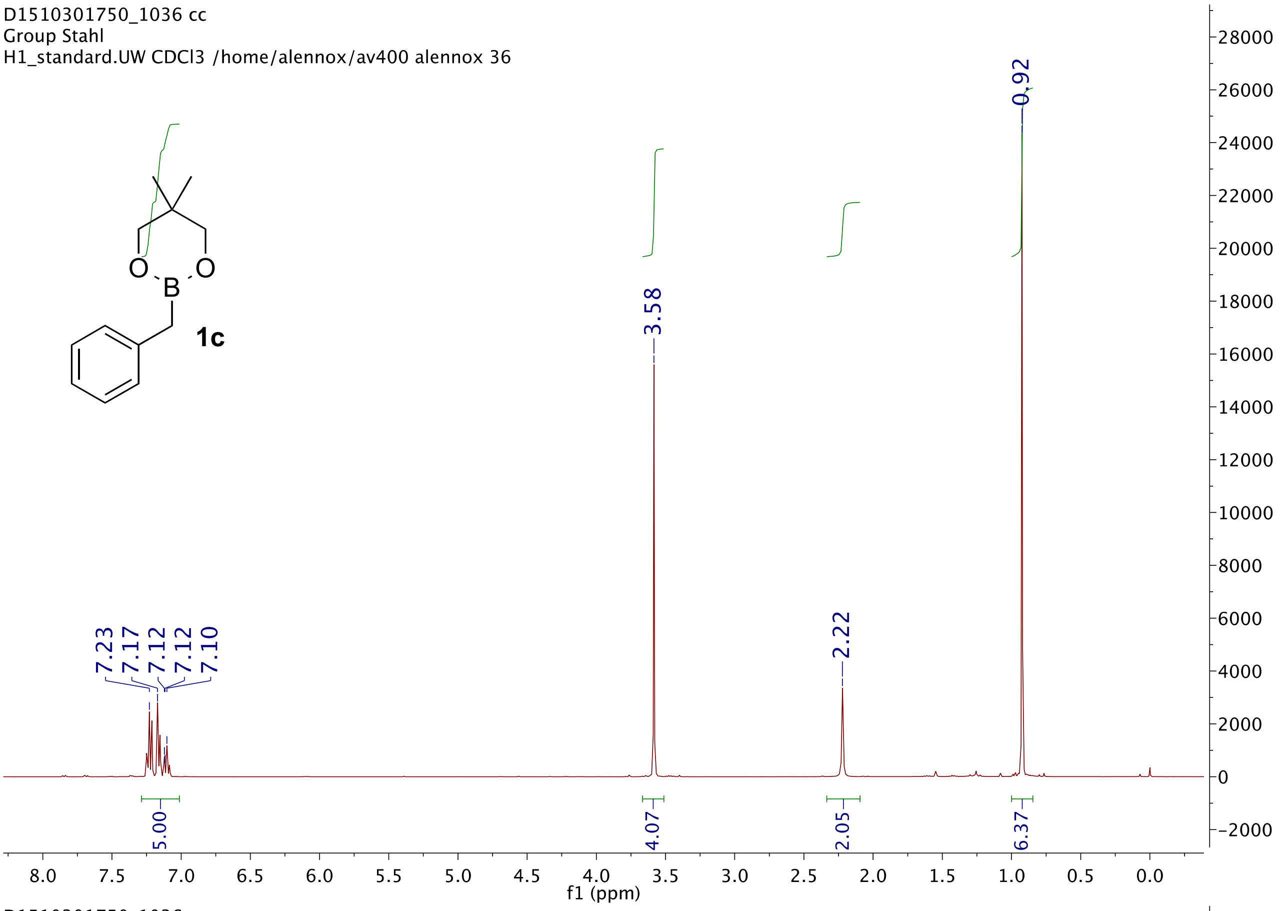
D1510301750_1036 cc
Group Stahl
H1_standard.UW CDCl3 /home/alennox/av400 alennox 36
1c
7.23
7.17
7.12
7.12
7.10
3.58
2.22
0.92
5.00
4.07
2.05
6.37
f1 (ppm)

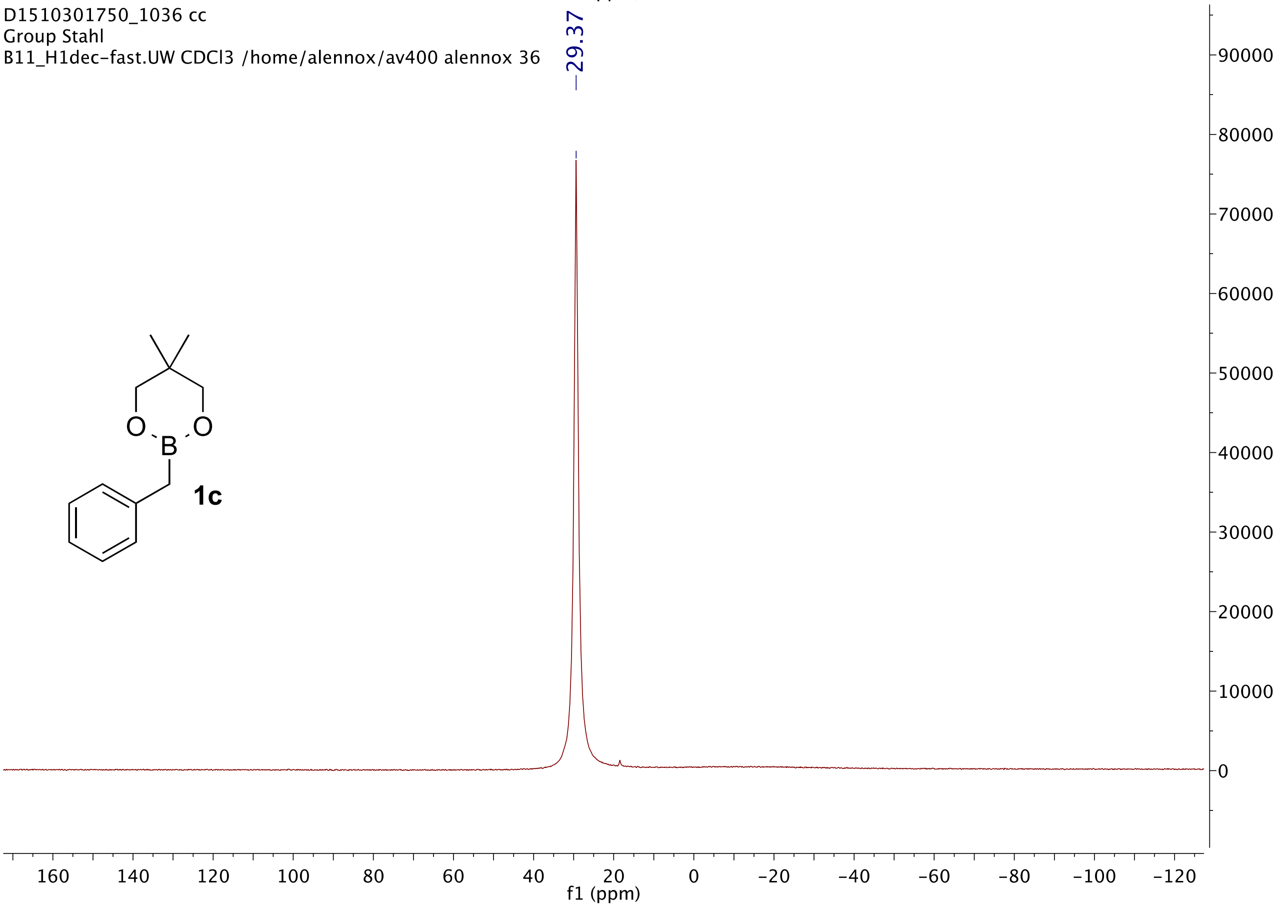
D1510301750_1036 cc
Group Stahl
B11_H1dec-fast.UW CDCl3 /home/alennox/av400 alennox 36
29.37
1c
f1 (ppm)

D1510301751_1040 cc
Group Stahl
H1_standard.UW CDCl3 /home/alennox/av400 alennox 37

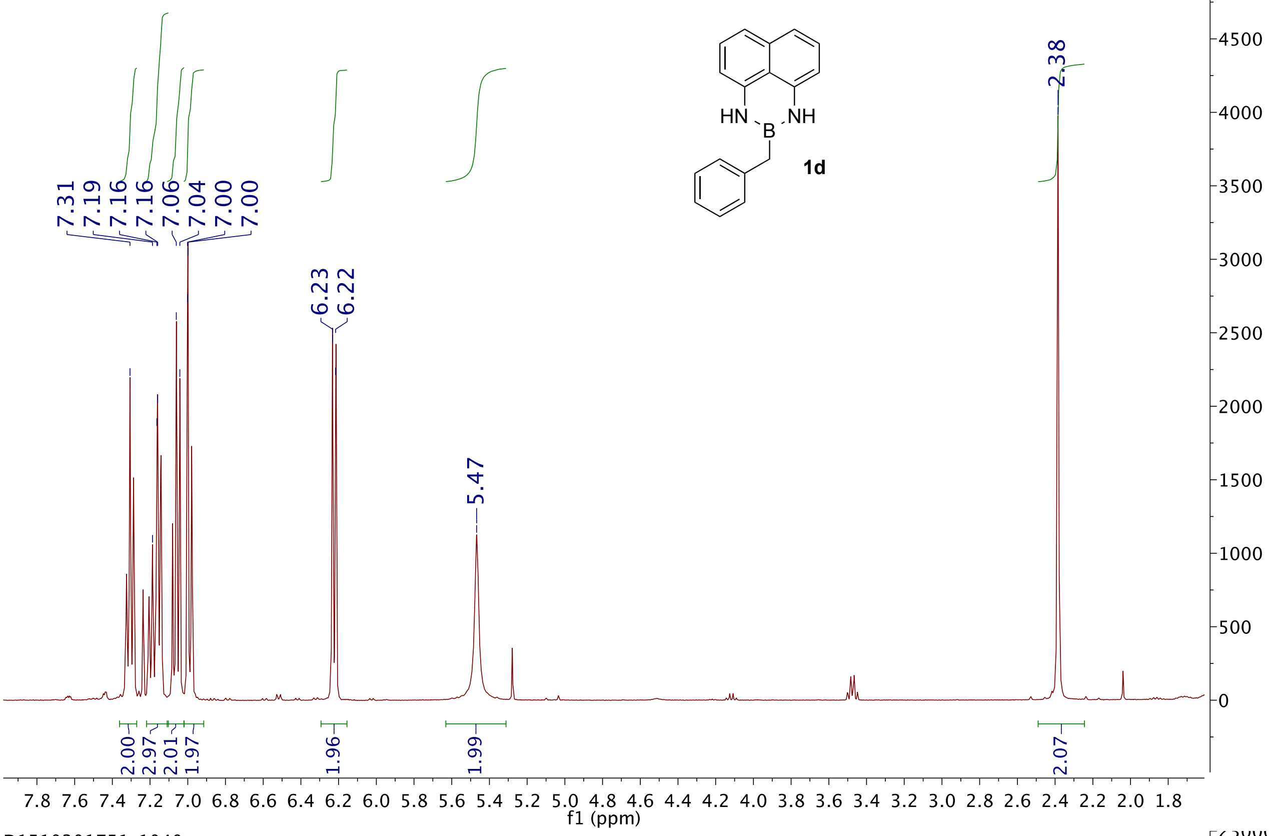

D1510301751_1040 cc
Group Stahl
B11_H1dec-fast.UW CDCl3 /home/alennox/av400 alennox 37

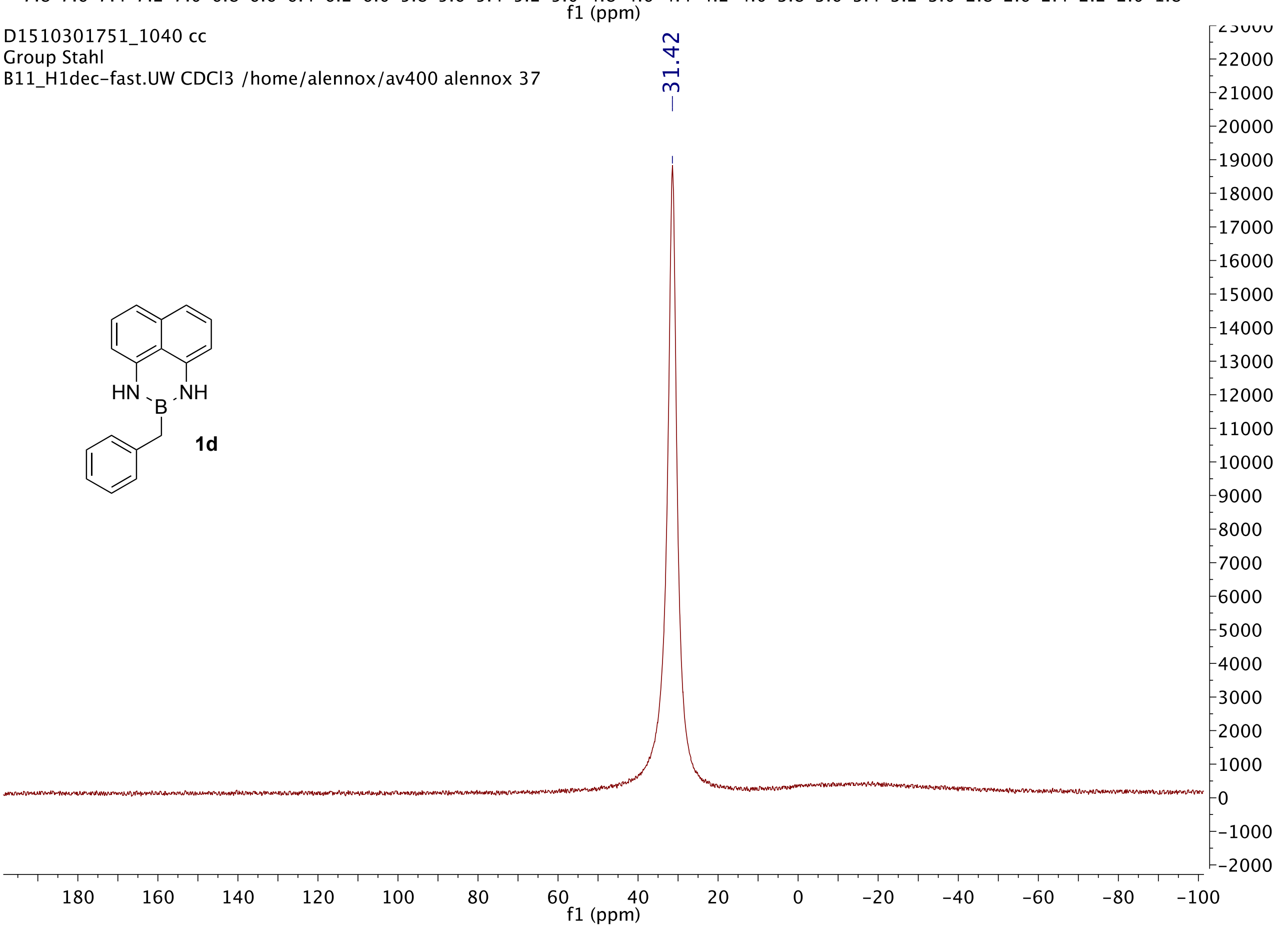

C1701051105_JEN1288.003.10.fid
Group Stahl
H1_standard.UW CDCl3 /home/jnutting/callisto jnutting 9

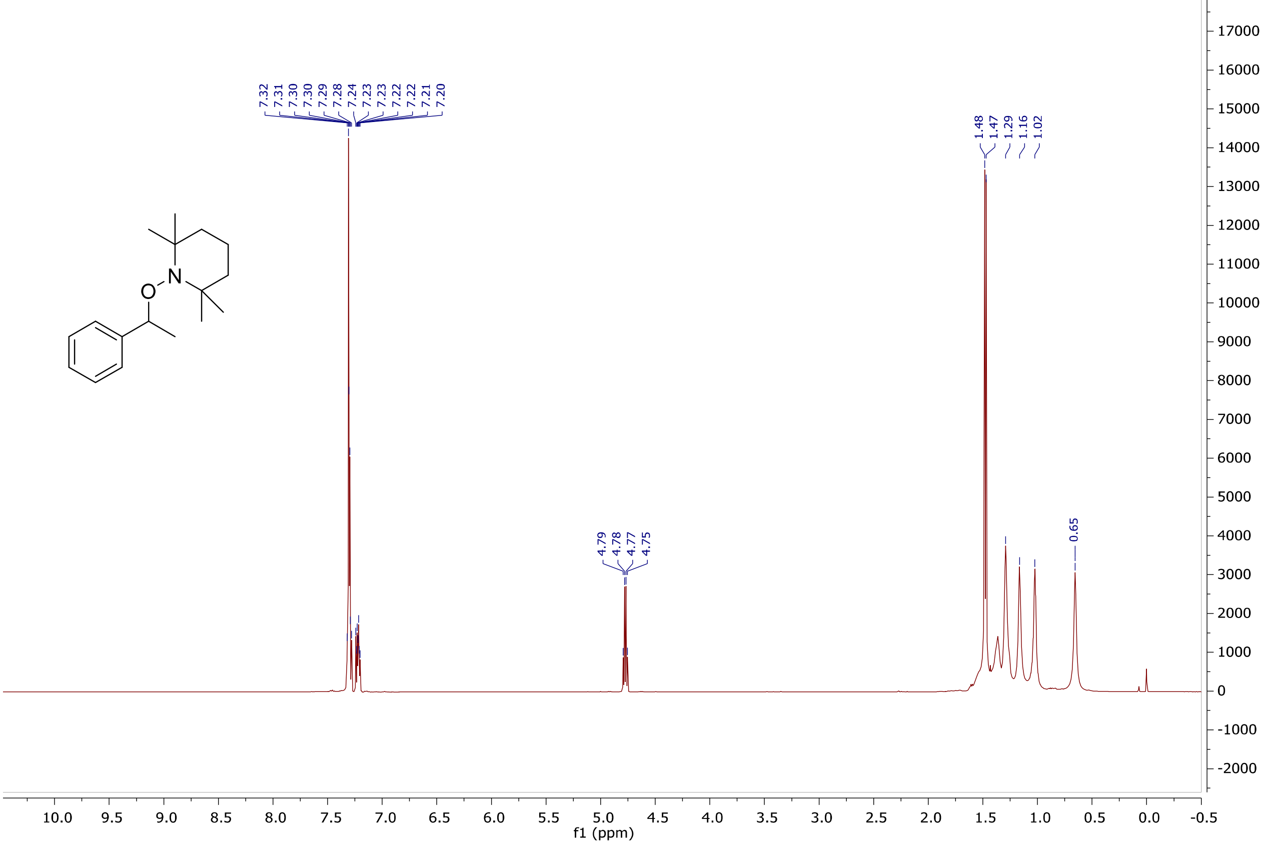

C1701051105_JEN1288.003.11.fid
Group Stahl
C13_H1dec.UW CDCl3 /home/jnutting/callisto jnutting 9

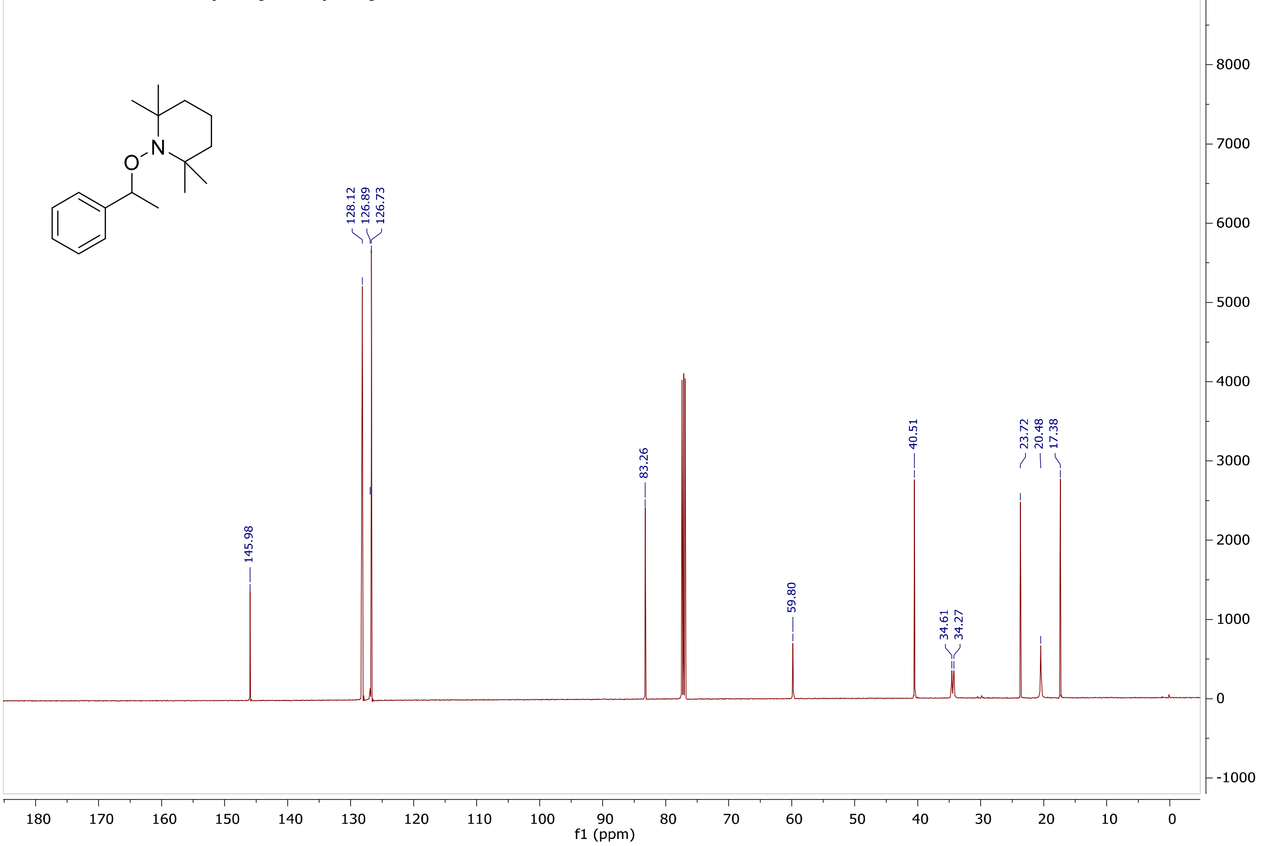

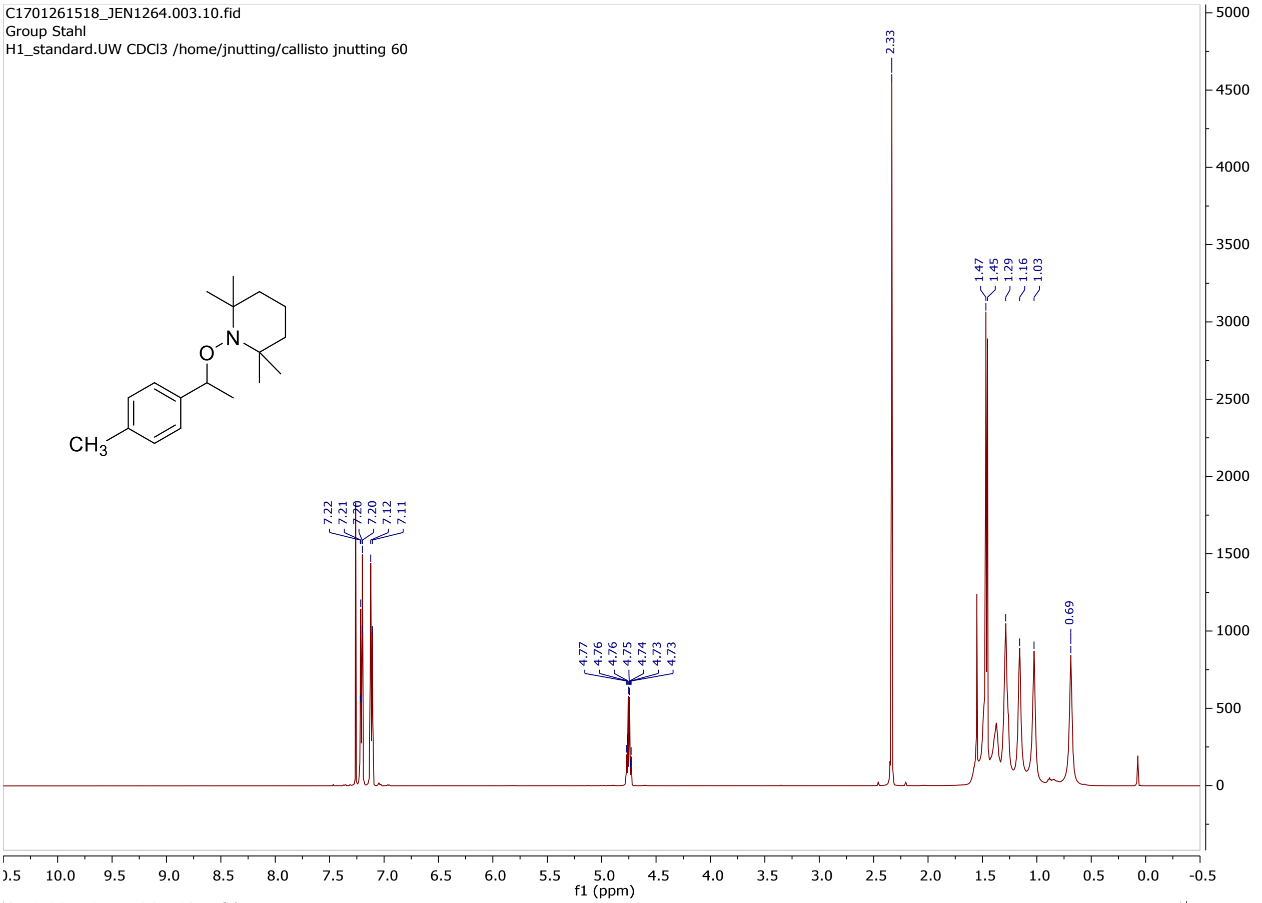
C1701261518_JEN1264.003.10.fid
Group Stahl
H1_standard.UW CDCl3 /home/jnutting/callisto jnutting 60
7.22
7.21
7.20
7.20
7.12
7.11
4.77
4.76
4.76
4.75
4.74
4.73
4.73
2.33
1.47
1.45
1.29
1.16
1.03
0.69
N
O
CH3
f1 (ppm)

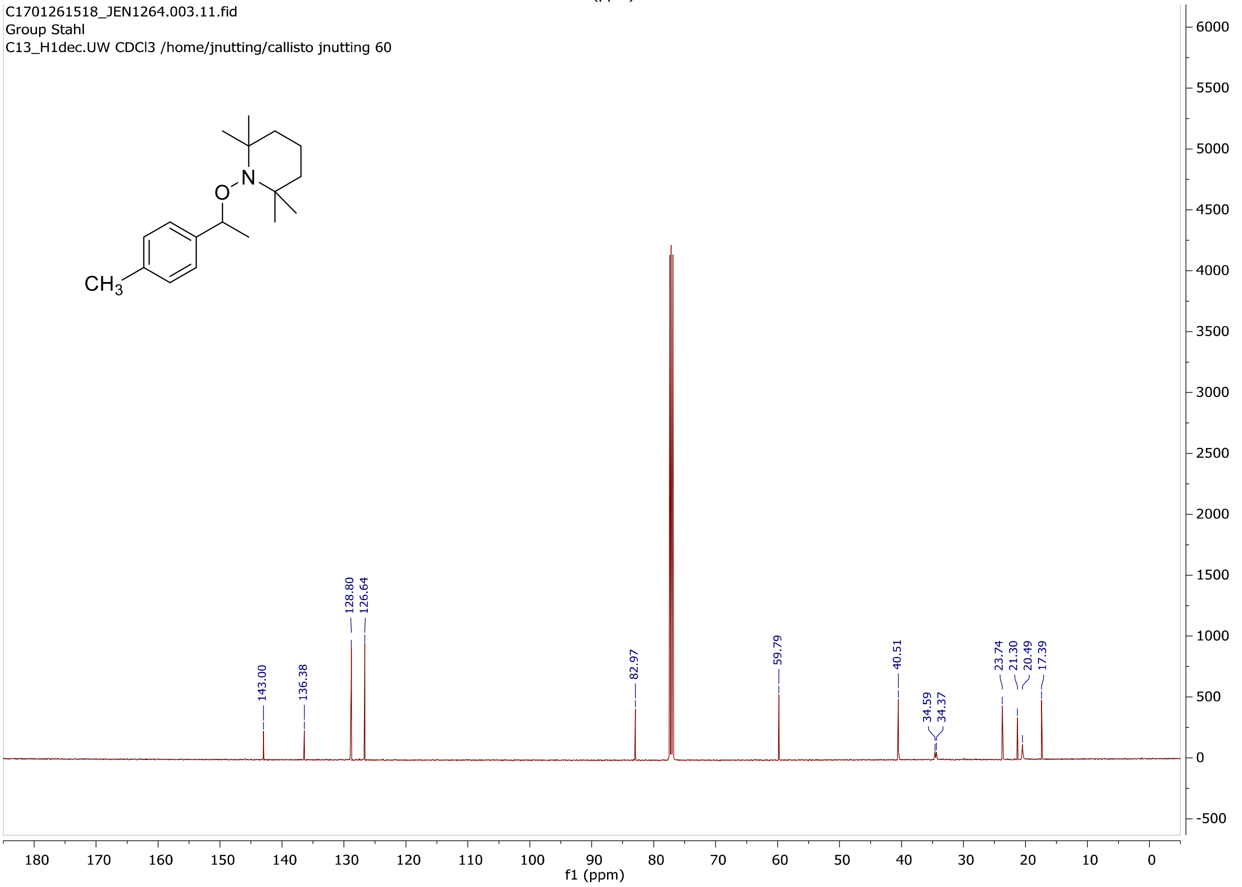
C1701261518_JEN1264.003.11.fid
Group Stahl
C13_H1dec.UW CDCl3 /home/jnutting/callisto jnutting 60
143.00
136.38
128.80
126.64
82.97
59.79
40.51
34.59
34.37
23.74
21.30
20.49
17.39
N
O
CH3
f1 (ppm)

D1701131114_JEN1295.001.10.fid
H1_standard.UW CDCl3 /home/jnutting/av400 jnutting 13

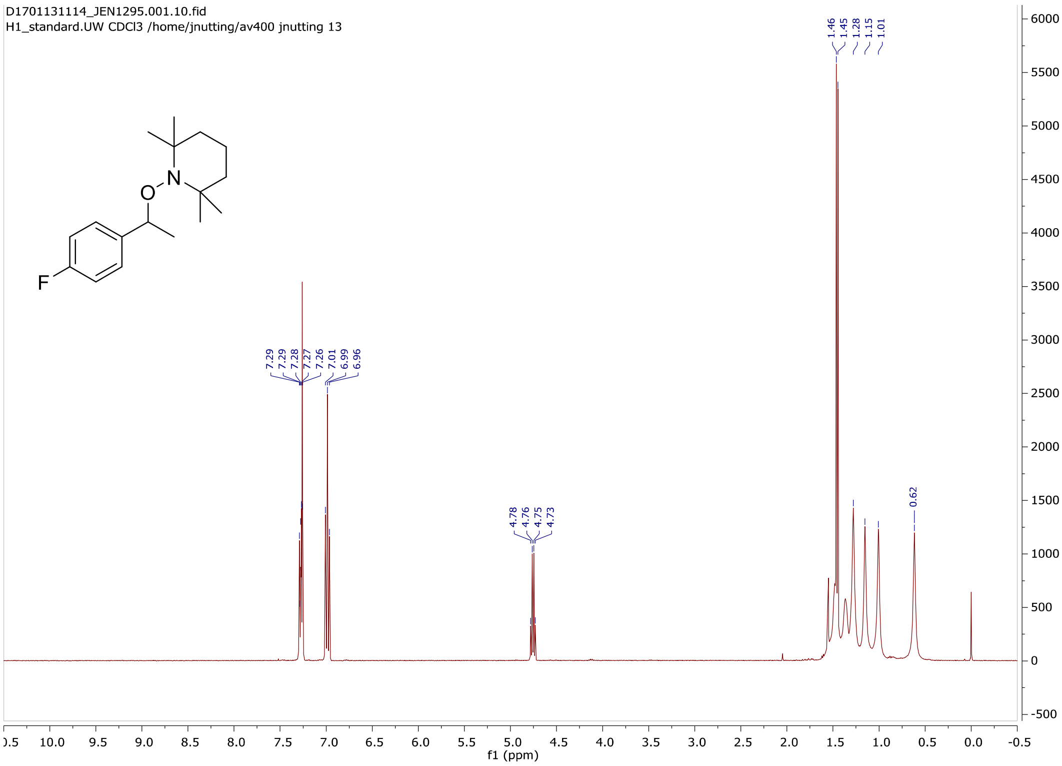

C1701261520_JEN1295.002.11.fid
Group Stahl
C13_H1dec.UW CDCl3 /home/jnutting/callisto jnutting 6

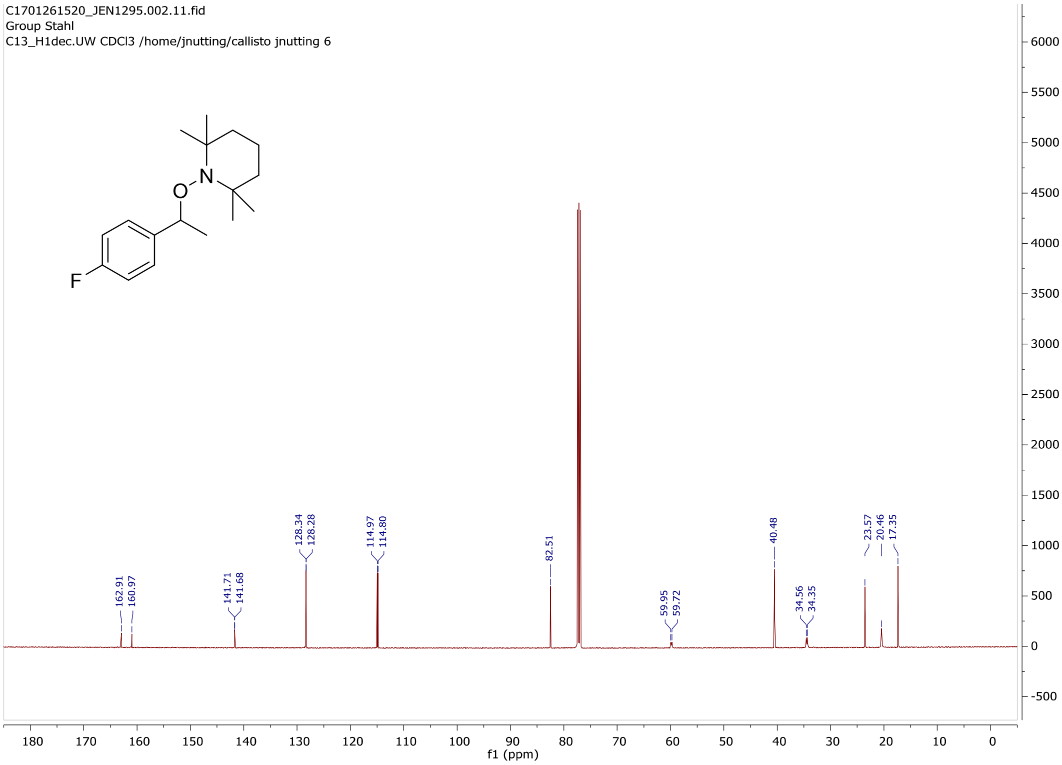

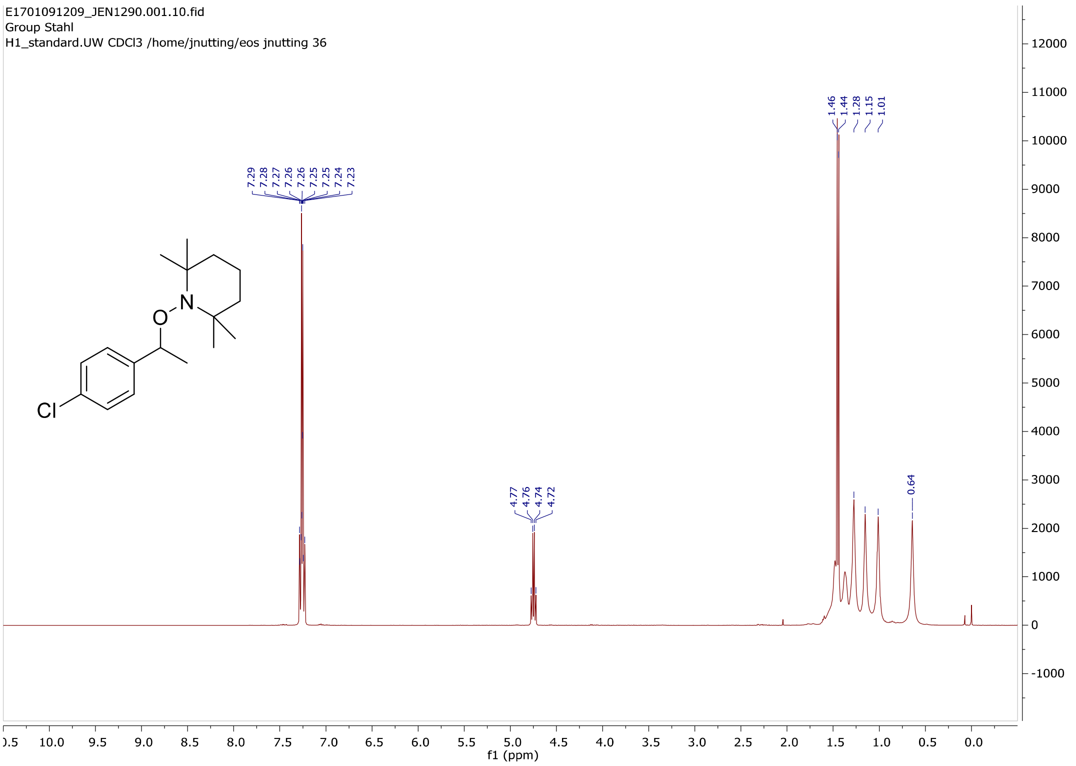
E1701091209_JEN1290.001.10.fid
Group Stahl
H1_standard.UW CDCl3 /home/jnutting/eos jnutting 36
7.29 7.28 7.27 7.26 7.26 7.25 7.25 7.24 7.23
4.77 4.76 4.74 4.72
1.46 1.44 1.28 1.15 1.01
0.64
Cl
O
N
f1 (ppm)

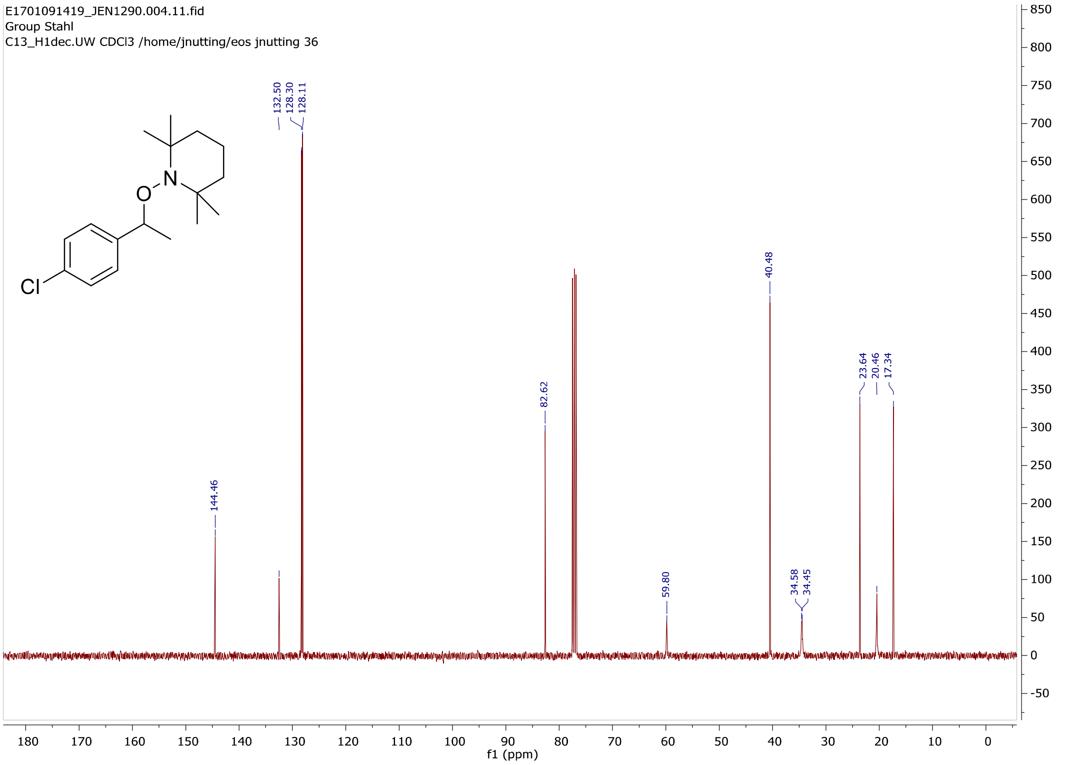
E1701091419_JEN1290.004.11.fid
Group Stahl
C13_H1dec.UW CDCl3 /home/jnutting/eos jnutting 36
132.50 128.30 128.11
144.46
82.62
59.80
40.48
34.58 34.45
23.64 20.46 17.34
Cl
O
N
f1 (ppm)

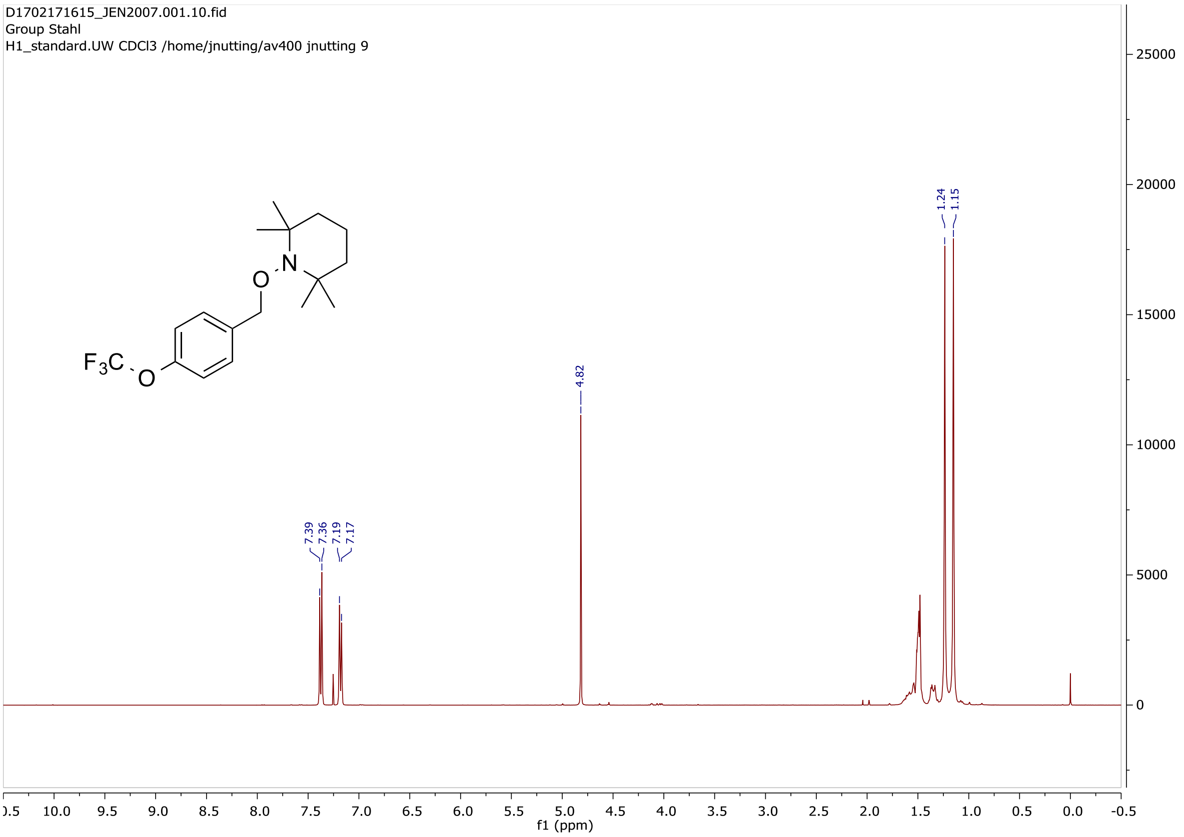
D1702171615_JEN2007.001.10.fid
Group Stahl
H1_standard.UW CDCl3 /home/jnutting/av400 jnutting 9
7.39
7.36
7.19
7.17
4.82
1.24
1.15
F3C
O
N
f1 (ppm)

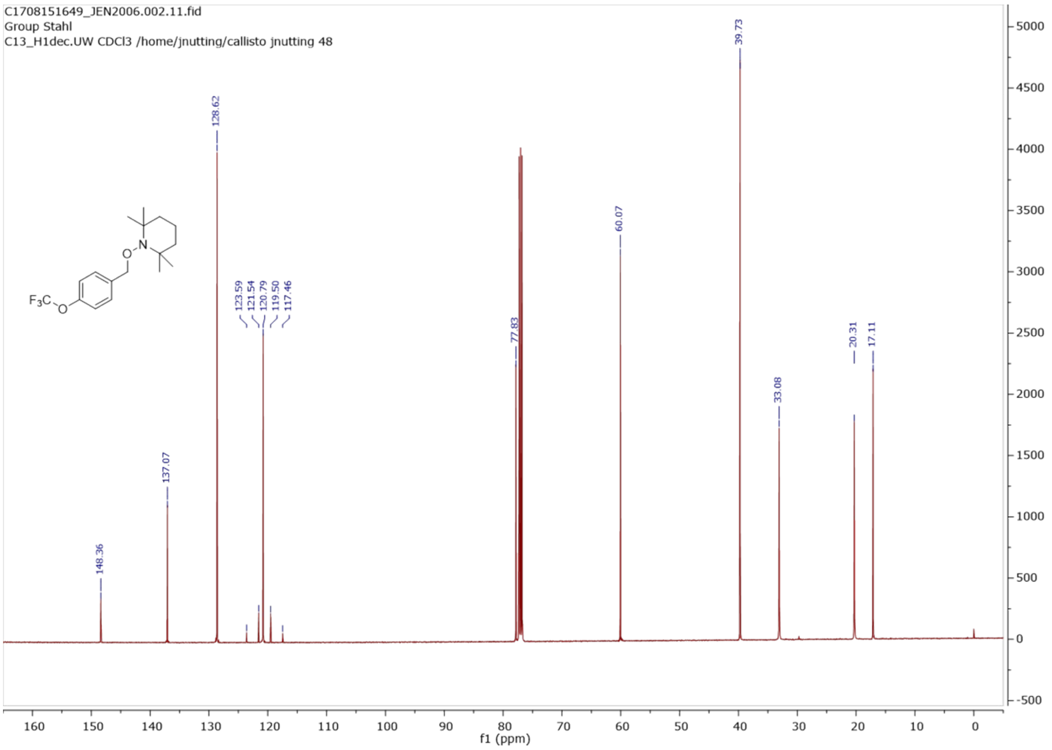
C1708151649_JEN2006.002.11.fid
Group Stahl
C13_H1dec.UW CDCl3 /home/jnutting/callisto jnutting 48
148.36
137.07
128.62
123.59
121.54
120.79
119.50
117.46
77.83
60.07
39.73
33.08
20.31
17.11
f1 (ppm)

12. References

1 K. Endo, M. Hirokami, K. Takeuchi and T. Shibata, *Synlett*, 2008, 3231–3233.
2 Q. I. Churches, J. F. Hooper and C. A. Hutton, *J. Org. Chem.*, 2015, **80**, 5428–5435.
3 C. J. Weiss, P. Das, D. L. Miller, M. L. Helm and A. M. Appel, *ACS Catal.*, 2014, **4**, 2951–2958.
4 T. Morofuji, A. Shimizu and J. I. Yoshida, *Chem. Eur. J.*, 2015, **21**, 3211–3214.
5 J. I. Yoshida, S. Suga, S. Suzuki, N. Kinomura, A. Yamamoto and K. Fujiwara, *J. Am. Chem. Soc.*, 1999, **121**, 9546–9549.
6 S. Suga, S. Suzuki and J. I. Yoshida, *Org. Lett.*, 2005, **7**, 4717–4720.
7 T. Morofuji, A. Shimizu and J. I. Yoshida, *J. Am. Chem. Soc.*, 2015, **137**, 9816–9819.
8 Y. Ashikari, Y. Kiuchi, T. Takeuchi, K. Ueoka, S. Suga and J. Yoshida, *Chem. Lett.*, 2014, **43**, 210–212.
9 S. Herold, S. Mohle, M. Zirbes, F. Richter, H. Nefzger and S. R. Waldvogel, *Eur. J. Org. Chem.*, 2016, **2016**, 1274–1278.
10 Q. Zhu, E. C. Gentry and R. R. Knowles, *Angew. Chem. Int. Ed.*, 2016, **55**, 9969–9973.
11 G. Zhang, H. Zeng, J. Wu, Z. Yin, S. Zheng and J. C. Fettinger, *Angew. Chem. Int. Ed.*, 2016, **55**, 14369–14372.
